# A Novel Definition and Grading Diagnostic Criteria for Tumour‐Type‐Specific Comprehensive Cachexia Risk

**DOI:** 10.1002/jcsm.13744

**Published:** 2025-03-21

**Authors:** Chunlei Hu, Minghua Cong, Chunhua Song, Hongxia Xu, Zengqing Guo, Fuxiang Zhou, Lan Zhou, Min Weng, Benqiang Rao, Li Deng, Kaiying Yu, Yongbing Chen, Ziwen Wang, Guotian Ruan, Ming Yang, Chenan Liu, Jiuwei Cui, Wei Li, Kunhua Wang, Zengning Li, Ming Liu, Tao Li, Junqiang Chen, Stephan von Haehling, Rocco Barazzoni, Hanping Shi

**Affiliations:** ^1^ Department of General Surgery, The First Hospital of Tsinghua University Tsinghua University Beijing China; ^2^ Department of Gastrointestinal Surgery, Department of Clinical Nutrition, Beijing Shijitan Hospital Capital Medical University Beijing China; ^3^ Key Laboratory of Cancer FSMP for State Market Regulation Beijing China; ^4^ Comprehensive Oncology Department, National Cancer Center/National Clinical Research Center for Cancer/Cancer Hospital Chinese Academy of Medical Sciences and Peking Union Medical College Beijing China; ^5^ Comprehensive Oncology Department, Hebei Cancer Hospital Chinese Academy of Medical Sciences and Peking Union Medical College Beijing China; ^6^ College of Public Health Zhengzhou University Zhengzhou China; ^7^ Army Medical Center of PLA Chongqing China; ^8^ Fujian Cancer Hospital Fuzhou China; ^9^ Zhongnan Hospital Wuhan University Wuhan China; ^10^ Yunnan Cancer Hospital Kunming China; ^11^ The First Affiliated Hospital Kunming Medical University Kunming China; ^12^ Department of Cardiology, Geriatric Cardiovascular Disease Research and Treatment Center The 82nd Group Army Hospital of PLA (252 Hospital of PLA) Baoding Hebei China; ^13^ Department of General Surgery, Beijing Friendship Hospital Capital Medical University Beijing China; ^14^ Department of Pharmacology, School of Basic Medical Sciences Capital Medical University Beijing China; ^15^ The First Affiliated Hospital Jilin University Changchun China; ^16^ Yunnan University Kunming China; ^17^ Department of Clinical Nutrition The First Hospital of Hebei Medical University Shijiazhuang China; ^18^ General Surgery Department, The Fourth Affiliated Hospital Harbin Medical University Harbin China; ^19^ Department of Radiotherapy, Sichuan Cancer Hospital & Institute, Sichuan Cancer Center, School of Medicine University of Electronic Science and Technology of China Chengdu China; ^20^ Department of Gastrointestinal Surgery First Affiliated Hospital of Guangxi Medical University Nanning China; ^21^ Department of Cardiology and Pneumology University Medical Center Göttingen Göttingen Germany; ^22^ German Centre for Cardiovascular Research (DZHK), Partner Site Göttingen Göttingen Germany; ^23^ Department of Medical, Surgical and Health Sciences University of Trieste Trieste Italy; ^24^ Beijing International Science and Technology Cooperation Base for Cancer Metabolism and Nutrition Beijing China; ^25^ National Clinical Research Center for Geriatric Diseases, Xuanwu Hospital Capital Medical University Beijing China

**Keywords:** cancer cachexia, comprehensive cancer cachexia risk, diagnostic criteria, solid tumour, tumour‐type‐specific diagnosis

## Abstract

**Background:**

The existing diagnostic criteria for cancer cachexia do not meet clinical needs. We aimed to establish novel comprehensive evaluation scales for cachexia specific to patients with solid tumours.

**Methods:**

This study included 12 651 patients (males: 6793 [53.7%]; females: 5858 [46.3%]; medium age: 58 [interquartile range:50/66] years; medium follow‐up duration: 24.16 [13.32/44.84] months; 4271 [33.8%] patients died; mean survival: 55.53 [95% confidence interval, 54.87/56.10] months; 3344 [26.4%], 4184 [33.1%] and 5123 [40.5%] patients with Stage I–II, III and IV tumour, respectively; derivation set: 10022, validation set: 2629 patients) with 14 types of solid tumours, including lung, gastric, liver, breast, oesophageal, cervical, bladder, pancreatic, prostate, ovarian, colorectal cancer, nasopharyngeal and endometrial carcinoma and cholangiocarcinoma, from an open and ongoing multicentre cohort study in China. Risk factors for cachexia, including tumour characteristics and nutritional parameters, were examined to develop diagnostic scales using Cox proportional hazards models and Kaplan–Meier analysis.

**Results:**

Ten nutrition items (body mass index, weight loss, intake reduction, physical activity function, fatigue, handgrip strength, anorexia, albumin level, albumin/globulin ratio and neutrophil/lymphocyte ratio) with different weighted scores were identified to construct a nutrition‐weighted scoring scale (NWSS) for nutrition risk. Tumour type and tumour burden status (tumour‐node‐metastasis stage and radical or non‐radical tumour) were determined to construct a disease‐weighted scoring scale (DWSS) for disease risk. A lumped scale (5 × 5 matrix) established using a five‐grade classification of nutrition and disease risk was used to determine a five‐grade classification of comprehensive cachexia risk: A, no cachexia risk (reference; lowest disease and nutrition risks); B, cachexia risk (hazard ratio [HR] = 4.517 [4.033/5.058]); C, pre‐cachexia (HR = 9.755 [8.73/10.901], medium survival = 21.21 months); D, cachexia (HR = 16.901 [14.995/19.049], medium survival = 11.61 months); and E, refractory cachexia (HR = 31.879 [28.244/35.981], medium survival = 4.83 months, highest disease and nutrition risks) (*p* < 0.001). Patients in Categories A–D benefited from nutrition therapy and anti‐tumour treatments to varying degrees. Patients in Category E were clinically refractory to nutrition therapy without prolonged survival compared with patients without nutrition therapy (medium survival, pre‐hospitalization nutrition therapy vs. hospitalization nutrition therapy vs. without nutrition therapy, 2.89 [1.91/3.88] vs. 4.04 [3.21/4.88] vs. 5.89 [4.73/7.04] months, *p* = 0.015) and anti‐tumour treatments without prolonged survival compared with patients receiving palliative care (medium survival, radical anti‐tumour treatments vs. adjuvant anti‐tumour treatments vs. palliative anti‐tumour treatments vs. and palliative care, 6.48 [4.42/8.53] vs. 6.48 [3.23/9.73] vs. 4.83 [4.22/5.44] vs. 2.70 [1.09/4.30] months, *p* = 0.263).

**Conclusion:**

We systematically developed a novel definition and grading diagnostic criteria for tumour‐type‐specific comprehensive cancer cachexia risk.

## 1 Introduction

Cachexia derives from the Greek word kachexia, meaning ‘bad condition’. It has multiple overlapping dimensional features including, but not limited to, loss of skeletal muscle and body weight, anorexia, physical activity function (PAF) impairment, fatigue and hyperinflammatory/catabolism and metabolic disorders, leading to increased treatment‐related toxicity, refractory to nutrition therapy and anti‐tumour treatments and reduced survival [1, 2, 3]. Cachexia may affect approximately 50%–80% patients with advanced cancer [4, 5, 6, 7]; of these, 20%–40% die due to cachexia [8]. However, the medical needs of cancer cachexia patients are far from being met [9, 10, 11]. Cachexia has been defined as disease‐related malnutrition (DRM) with an inflammatory component [12], which cannot be fully reversed by nutrition therapy due to anabolic resistance owing to hyperinflammatory/catabolism and metabolic disorders [12, 13, 14, 15]. Malnutrition without hyperinflammatory/catabolism and metabolic disorders such as malnutrition primarily related to low nutrient intake can be fully reversed by appropriate nutrition therapy [12].

A widely recognized 2011 expert consensus (termed Consensus 2011 hereafter) proposed that cancer cachexia is a multifactorial syndrome characterized by an ongoing skeletal muscle mass loss (with or without loss of fat mass) that cannot be fully reversed by conventional nutritional support and leads to progressive functional impairment [2]. Consensus 2011 further proposed the concept of grading cachexia (pre‐cachexia, cachexia and refractory cachexia). Pre‐cachexia may further develop to cachexia; these patients need nutritional status monitoring and preventive intervention. Patients with cachexia need multimodal management according to phenotype and clinical characteristics, with prioritization of reversible contributory factors, to improve responses to nutrition therapy and prevent or delay cachexia progression. Patients with refractory cachexia are clinically unresponsive to nutrition therapy and anti‐tumour treatments; potential risks of medical nutritional therapy are likely to outweigh the benefit, and symptom palliation and psychosocial support are primarily recommended. Consensus 2011 further noted that the risk of cachexia progression varies and depends on disease progression, nutritional deterioration and responsiveness to nutrition therapy and anti‐tumour treatments; and a refined grading of cachexia should be also based on clinical outcomes, including death risk and responses to nutrition therapy and anti‐tumour treatments [1, 3, 16].

The concept of grading cachexia greatly helps clinicians in clinical practice; however, defects remain. First, because robust indicators of hyperinflammatory/catabolism and metabolic disorders are incompletely defined in clinical screening approaches, Consensus 2011 only developed basic screening and diagnostic criteria for cachexia diagnosis based on weight loss, low body mass index (BMI) and skeletal muscle depletion [2]. It did not further define operable cachexia grading. However, several studies found that criteria for cachexia, based on sarcopenia and BMI‐adjusted‐weight loss only [2, 15, 16, 17], could not distinguish pre‐cachexia from non‐cachexia [18, 19]. Second, Consensus 2011 states that the risk of cachexia is determined by both nutritional status and disease characteristics. However, to our knowledge, no study has effectively quantified the impact of disease characteristics on cachexia and cachexia progression risk. Third, Consensus 2011 suggests that cachexia classification should be based on clinical outcomes including the survival time and the responses to nutrition therapy and anti‐tumour treatments. However, no study has verified the predictive value of their classification of cachexia on responses to nutrition therapy and anti‐tumour treatments. Overall, the existing diagnostic criteria for cancer cachexia do not fully meet clinical needs, and the development of more refined diagnostic tools for cachexia with comprehensive consideration of disease characteristics, nutritional parameters and clinical outcomes is imperative.

Therefore, we aimed to establish novel comprehensive evaluation scales for cachexia specific to patients with solid tumours based on a large multidimensional database from an ongoing multicentre cohort study in China, the Investigation on Nutrition Status and its Clinical Outcome of Common Cancers (INSCOC) [20].

## 2 Methods

### 2.1 Study Population and Design

The INSCOC is an open and ongoing cohort study aimed to evaluate associations among multidimensional disease and nutritional parameters and outcomes in patients with cancer. In March 2020, we began designing novel comprehensive diagnostic scales for cachexia with approval from INSCOC's workshop and the human research ethics committees of all participating hospitals. In INSCOC, only the data reviewed by a third team can be released and used for statistical analysis, and the follow‐up survival data have been annually updated and released for analysis since 2019. The study retrospectively included 12 651 patients enrolled between January 2013 and January 2020. To reduce potential bias of homogenized data using internal validation, we intentionally selected a subset (10 022 patients) released in 2019 as the derivation set and a subset (2629 patients) released in 2020 as the validation set, rather than randomly paired patients from the overall samples (released in 2019 and 2020).

**FIGURE 1:**
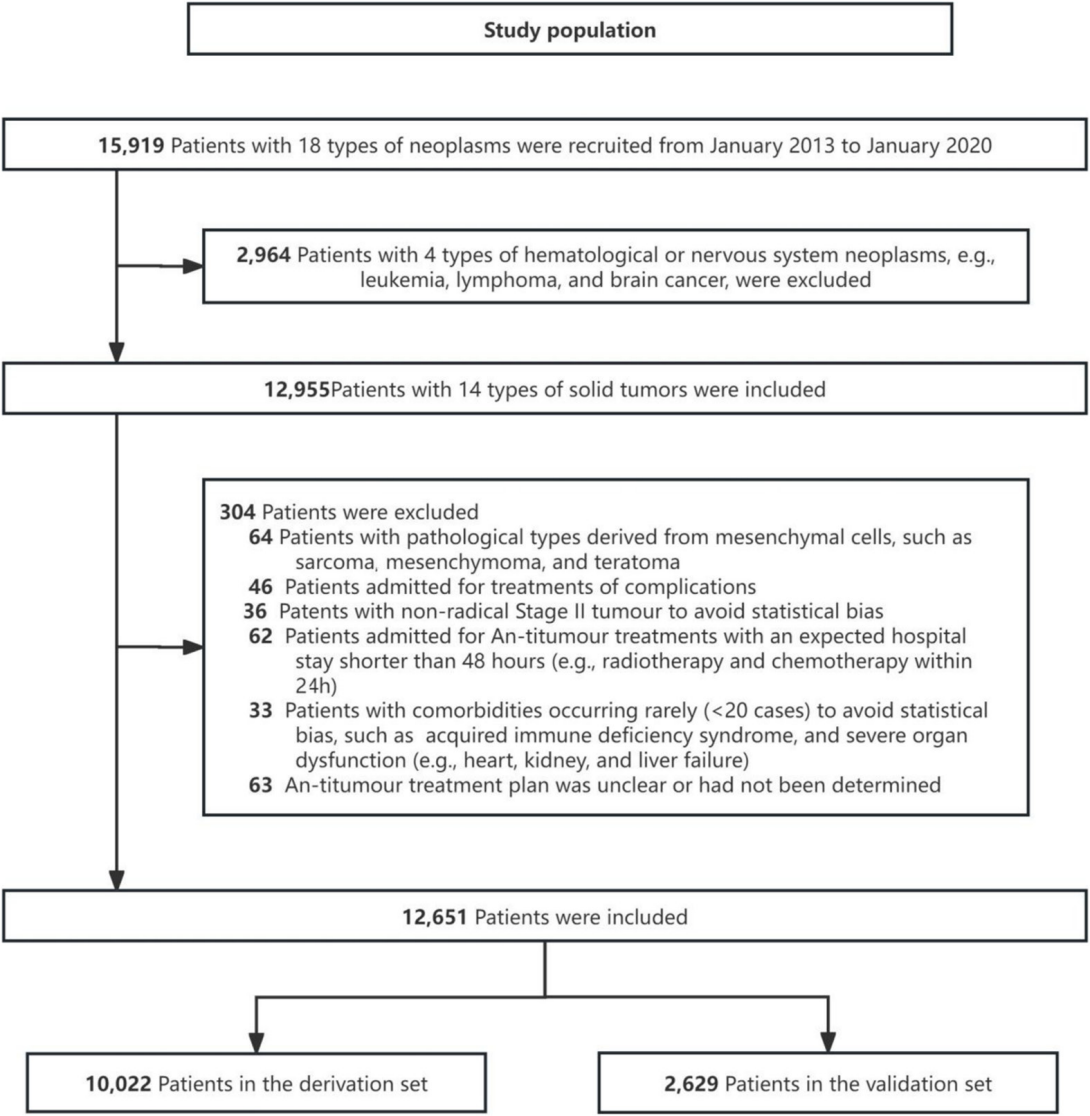
The flow chart of study population.

Inclusion criteria were (1) patients aged ≥ 18 years; (2) patients with one or more of 14 types of solid tumours (with pathological types derived from mucosal, acinar and ductal epithelial cells), including lung, gastric, liver, breast, oesophageal, cervical, bladder, pancreatic, prostate, ovarian, colorectal cancer, nasopharyngeal and endometrial carcinoma and cholangiocarcinoma; and (3) patients admitted for scheduled anti‐tumour treatments or palliative care. Exclusion criteria were (1) patients with pathological types derived from mesenchymal cells, including sarcoma, mesenchymoma and teratoma, and (2) patients with undetermined treatment plan. Figure 1 depicts the study population.

### 2.2 Data Characteristics

The available cachexia‐related disease and nutrition indicators in the INSCOC database were selected based on a thorough review of previous studies [1, 3, 8, 9, 21].

Nutritional/functional indicators included weight loss [22], food intake reduction, anorexia, BMI, skeletal muscle mass, handgrip strength (HGS), laboratory indicators (albumin/globulin ratio [AGR], neutrophil/lymphocyte ratio [NLR] and albumin and haemoglobin levels), functional effects (PAF and fatigue) [23], sarcopenia [24] and cachexia as per Consensus 2011 [2]. Disease indicators included tumour characteristics (tumour type, tumour node metastasis [TNM], tumour recurrence and metastasis, tumour‐bearing status [resected or unresected tumours]) and cachexia‐associated comorbidities [25]. All variables were fixed during the first baseline survey and are briefly listed in Table 1 and explicated in detail in Supporting Information S1.

**TABLE 1:** Patients' general medical and demographic characteristics.

Variables		Derivation set (*n*/%) or (medium [IQR])	Validation set (*n*/%) or (medium [IQR])	*p*
General clinical characteristics				
Gender	Men	5338/53.3%	1455/55.3%	0.057^a^
	Women	4684/46.7%	1174/44.7%	
Age (years)		58 (50/66)	59 (50/66)	0.43^b^
Follow‐up time (months)		24.89 (13.61/46.46)	22.42 (12.77/33.75)	< 0.001^b^
Mean survival (months)		55.23	56.49	0.024^c^
Nutrition therapy (NT)	Without NT	7267/73.9%	1906/73.3%	0.002^a^
	Pre‐hospitalization NT	1334/13.6%	413/15.9%	
	Hospitalization NT	1230/12.5%	282/10.8%	
Anti‐tumour treatments (ANTs)	Radical ANTs	3394/33.9%	825/31.4%	< 0.001^a^
	Adjuvant ANTs	2554/25.5%	785/29.9%	
	Palliative ANTs	3897/38.9%	972/38.5%	
	Palliative care	177/1.8%	47/1.8%	
Radical anti‐tumour treatments	No	6628/66.1%	1804/68.6%	0.016^a^
	Yes	3394/33.9%	825/31.4%	
Tumour characteristics				
Breast cancer	Yes	1580/15.8%	364/13.8%	0.014^a^
Nasopharyngeal carcinoma	Yes	1032/10.3%	193/7.3%	< 0.001^a^
Endometrial carcinoma	Yes	105/1.0%	37/1.4%	0.119^a^
Cervical cancer	Yes	381/3.8%	89/3.4%	0.315^a^
Prostatic cancer	Yes	101/1.0%	51/1.9%	< 0.001^a^
Ovarian cancer	Yes	239/2.4%	62/2.4%	0.937^a^
Bladder cancer	Yes	119/1.2%	32/1.2%	0.9^a^
Colorectal cancer	Yes	1858/18.5%	475/18.1%	0.579^a^
Gastric cancer	Yes	1257/12.5%	244/9.3%	< 0.001^a^
Oesophageal cancer	Yes	659/6.6%	258/9.8%	< 0.001^a^
Liver cancer	Yes	271/2.7%	88/3.3%	0.077^a^
Lung cancer	Yes	2268/22.6%	691/26.3%	< 0.001^a^
Pancreatic cancer	Yes	137/1.4%	38/1.4%	0.759^a^
Cholangiocarcinomas	Yes	58/0.6%	17/0.6%	0.686^a^
Tumour‐node‐metastasis stage	I	1123/11.2%	135/5.1%	< 0.001^a^
	II	1782/17.8%	304/11.6%	
	III	2983/29.8%	1201/45.7%	
	IV	4134/41.2%	989/37.6%	
Distant organ metastasis (numbers, *n*)	0	7088/70.7%	2042/77.6%	< 0.001^a^
	1	1636/16.3%	325/12.4%	
	≥ 2	1298/13.0%	262/10.0%	
Tumour recurrence	No	7651/76.3%	2350/89.4%	< 0.001^a^
	Yes	2371/23.7%	279/10.6%	
Existing focus	No	2554/25.5%	785/29.9%	< 0.001^a^
	Yes	7468/74.5%	1844/70.1%	
Comorbidity				
Cirrhosis	Yes	109/1.1%	24/0.9%	0.434^a^
Chronic hepatitis	Yes	454/4.5%	115/4.4%	0.732^a^
Cerebral stroke	Yes	71/0.7%	17/0.6%	0.734^a^
COPD	Yes	113/1.1%	37/1.4%	0.238^a^
Tuberculosis	Yes	34/0.3%	20/0.8%	0.03^a^
Myocardial infarction	Yes	31/0.3%	4/0.2%	0.172^a^
Diabetes	Yes	816/8.1%	227/8.6%	0.414^a^
Hypertension	Yes	1756/17.5%	527/20%	0.003^a^
Coronary heart disease	Yes	424/4.2%	105/4%	0.589^a^
Chronic kidney disease	Yes	22/0.2%	7/0.3%	0.656^a^
Nutritional/functional characteristics				
Albumin (g/L)		39.40 (35.90/42.60)	40.00 (36.35/43.60)	< 0.001^b^
AGR (ratio)		1.38 (1.19/1.57)	1.44 (1.24/1.64)	< 0.001^b^
Haemoglobin (g/L)		126.0 (112.0/138.0)	126.0 (111.0/138.0)	0.971^b^
NLR (ratio)		2.48 (1.67/3.99)	2.58 (1.69/4.11)	0.229^b^
C‐reactive protein (mg/L)		3.70 (2.04/16.85)	3.40 (1.21/14.57)	0.001^b^
BMI (kg/m^2^)		22.66 (20.40/24.97)	22.39 (20.13/24.65)	< 0.001^b^
MAC (%)		94.41 (87.27/101.82)	94.41 (86.55/101.82)	0.482^b^
TSF (%)		132.53 (96.39/183.01)	103.03 (72.36/144)	< 0.001^b^
MAMC (%)		87.72 (80.04/95.6)	88.38 (80.47/97.08)	0.057^b^
FFMI (%)		93.81 (87.84/100.24)	92.40 (85.35/98.53)	0.01^b^
%WL within 1 month		0 (−3.33/0)	0 (−3.23/0)	0.686^b^
%WL within 6 month		−1.61 (−7.69/0)	0 (−6.25/0)	< 0.001^b^
HGS (kg)		23.80 (17.70/30.80)	25.00 (17.20/31.20)	0.113^b^
Intake reduction (sore)		0 (0/1)	0 (0/1)	0.435^b^
E‐PAF (score)		6 (5/8)	5(5/7)	< 0.001^b^
Fatigue (score)		4 (3/6)	4 (3/6)	0.861^b^
Anorexia (score)		0 (0/0)	0 (0/1)	< 0.001^b^
Karnofsky score (score)		90 (80/90)	90 (80/100)	< 0.001^b^
P‐PAF, (score)		0 (0/1)	0 (0/1)	< 0.001^b^
Sarcopenia	Yes	1361/13.6%	364/13.9%	0.73^a^
PG‐SGA category	A	4851/48.4%	1073/40.8%	< 0.001^a^
	B	3111/31.0%	853/32.5%	
	C	2060/20.6%	703/26.7%	

*Note:* The included data are illustrated in detail in Supporting Information S1.

Abbreviations: AGR, albumin/globulin ratio; BMI, body mass index; COPD, chronic obstructive pulmonary disease; E‐PAF, fatigue and anorexia, the raw score of the physical activity function, fatigue, and anorexia domains in the quality of life instrument designed by the European Organization for Research and Treatment of Cancer (EORTC QLQ‐C30); FFMI, fat free mass index; HGS, handgrip strength; MAC, mid‐arm circumference; MAMC, mid‐arm muscle circumference; NLR, neutrophil/lymphocyte ratio; P‐PAF, physical activity function component of the Patient‐Generated Subjective Global Assessment (PG‐SGA); TSF, triceps skin fold; WL, weight loss.

^a^ Chi‐square test.

^b^ Kruskal–Wallis test.

^c^ Log‐rank tests.

### 2.3 Anti‐tumour Treatment and Nutrition Therapy Subgroups

Patients were divided into four groups according to goals of anti‐tumour treatments, determined by patients' medical histories and schedules: radical anti‐tumour treatments (radical surgery and radiotherapy), adjuvant anti‐tumour treatments (adjuvant chemotherapy, radiotherapy, molecular targeted therapy, immunotherapy and endocrine therapy after radical surgery), palliative anti‐tumour treatments (palliative surgery, chemotherapy, radiotherapy, molecular targeted therapy and immunotherapy in patients with non‐radical tumours) and palliative care (symptomatic care only for relieving pain and improving quality of life).

Patients were divided into three groups according to nutrition therapy status: pre‐hospitalization nutrition therapy (pre‐emptive nutritional pre‐habilitation; received nutrition therapy ≥ 1 week before hospitalization, including enteral nutrition [EN], total parenteral nutrition [TPN] and parenteral + enteral nutrition [PN + EN]), hospitalization nutrition therapy (received nutrition therapy ≥ 1 week after hospitalization) and not receiving nutrition therapy.

### 2.4 Outcomes

Survival data, including time and cause of death, were acquired biannually via inpatient medical visit records and telephone interviews. Overall survival was defined as the interval between the date of the first survey and death.

### 2.5 Statistical Analyses

Statistical analyses were performed using SPSS (v.22.0) and R (v.2.15.2) statistical software. Descriptive statistics were analysed using chi‐square tests and Kruskal–Wallis tests, as appropriate. Univariate and multivariate Cox proportional hazards models were used to generate hazard ratios (HRs) and associated 95% confidence intervals (CIs). The receiver operating characteristic (ROC) curves and areas under the curves (AUCs) were used to evaluate the diagnostic value of the indicators and scales. The survival time was evaluated by the Kaplan–Meier method and compared with log‐rank tests. If median survival was not available, mean survival is presented instead. Statistical significance was established at *p* < 0.05 (two‐sided) for analyses, except for multivariate Cox proportional hazards models, for which it was established at *p* < 0.10.

### 2.6 Establishment of Scales

#### 2.6.1 Step 1: Screening Optimum Indicators for Cachexia Risk

We used the overall death risks (HRs) to screen optimal nutrition and disease indicators for cachexia followed by previous studies [17].

Many studies include sarcopenia as an important component for diagnosis of cachexia [2, 11, 17]. Sarcopenia is defined as loss of skeletal muscle mass and function. However, our data demonstrated that skeletal muscle mass [2, 26], and HGS and PAF decline independently impact death risk. Hence, we intend to include them independently in further analyses. Because accurate body composition analysis is expensive and inconvenient in nutrition screening jobs, we included easily available BMI as a surrogate marker for skeletal muscle mass, as previously recommended for diagnosis of cachexia [2, 17].

#### 2.6.2 Step 2: Establishment of a Nutrition‐Weighted Scoring Scale (NWSS)

##### 2.6.2.1 Optimal indicators and Their Weighted Scores

Ten optimal nutrition items were selected by the univariate and multivariate Cox proportional hazards analyses. For effectiveness and simplicity, nutrition indicators were classified into three hierarchical categories to evaluate the severity of cachexia: normal, moderate and severe. Raw grades of the hierarchical variables with similar HRs and no significant differences were combined together into three new joint classifications. Albumin and AGR were characterized using clinical routine cut‐off values. NLR and HGS have no clinical routine cut‐off values and were therefore stratified using Youden indices calculated from the ROC curves (NLR) or quartiles by sex and age groups (HGS) for adult males (aged 18–64 years), adult females, elderly males (≥ 65 years) and elderly females.

The weighted scores (with approximate rounding) of the optimal indicators were determined by their HRs (the greater the HR, the higher the score assigned) using Cox proportional hazards models. These items included low albumin and AGR (with the highest weighted scores: 0, 2, 4); followed by anorexia, increased NLR and intake loss (0, 2, 3), low BMI and PAF (0, 1, 3); and weight loss, fatigue and low HGS (with the lowest weighted scores; 0, 1, 2). Then, the nutrition‐weighted scoring scale (NWSS) was established using a summation method.

##### 2.6.2.2 NWSS Classification (Nutrition Risk)

**FIGURE 2:**
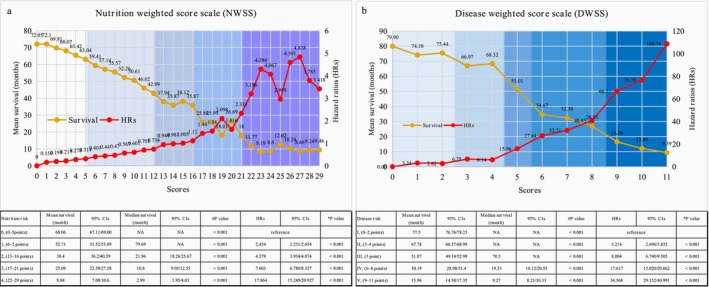
Hazard ratios (HRs) and mean survivals using different scores and classifications. Notes: Line graphs representing the relationships between scores derived from the (a) nutrition‐weighted scoring scale (NWSS) and (b) disease‐weighted scoring scale (DWSS) in regard to overall survival. Red lines represent increased estimated hazard ratios (HRs), and brown lines represent decreased survival (months) associated with increased scores. Different background colours represent NWSS (nutrition risk) and DWSS (disease risk) classifications. 95% CIs, 95% confidence intervals. #*p* value for survival (log‐rank tests), *p values for the HRs (Cox proportional hazards model [sample method]).

The scores with similar HRs and survivals (without significant difference) (0–29 points) were combined into a five‐grade classification of nutrition risk (Figure 2) [17]. Table 2 presents the final NWSS. Supporting Information S2 depicts the process of establishing the NWSS.

**TABLE 2:** The nutrition‐weighted scoring scale (NWSS).

Items^a^	Cut‐off values and weighted scores in the classifications
Albumin^b^ (g/L)	≥ 35 = 0 point; 28–34.9 = 2 points; < 28 = 4 points
Albumin/globulin ratio^c^ (ratio)	≥ 1.3 = 0 point; 1–1.29 = 2 points; 0–0.99 = 4 points
Body mass index^d^ (kg/m^2^)	≥ 24 = 0 point; 18.5–23.9 = 1 point; < 18.5 = 3 points
Neutrophils^e^ /lymphocytes ratio (ratio)	0–3.49 = 0 point; 3.50–6.80 = 2 points; > 6.80 = 3 points
Hand grip strength^f^ (kg)	< 65 y: male ≥ 31 = 0 points; 25–30.9 = 1 point; ≤ 24.9 = 2 points; female ≥ 20 = 0 points; 15–19.9 = 1 point; ≤ 14.9 = 2 points ≥ 65 y: male ≥ 26 = 0 points; 20–25.9 = 1 point; ≤ 19.9 = 2 points; female ≥ 18 = 0 points; 12–17.9 = 1; ≤11.9 = 2 points
Weight loss^g^	Within 1 month: no weight loss or weight loss < 2% = 0 point; 2%–4.9% = 1 point; ≥ 5% = 2 points Within 6 months: no weight loss or weight loss < 2% = 0 point; 2%–9.9% = 1 point; ≥ 10% = 2 points
Intake reduction^h^	Within 1 month: no reduced intake = 0 point; reduced intake ≤ 25% = 2 points; reduced intake > 25% = 3 points
Physical active function^i^	No difficulty carrying heavy objects (approximately 20 kg) or walking long distances (approximately 2 km) = 0 point Difficulty carrying heavy objects (approximately 20 kg) or walking long distances (approximately 2 km) = 1 point Difficulty walking short distances (within 1 km); or needing to stay in bed or a chair during the day = 3 points
Fatigue^j^	Do not feel weak and tired; or need a rest = 0 point Have two of the following lesions: weak, tired, frequently need a rest = 1 point Have three of the following lesions: weak, tired, frequently need a rest = 2 points
Anorexia^k^	No anorexia = 0 point; a bite anorexia = 2 points; quiet and very anorexia = 3 points

*Note:* The classification of the NWSS (0–29 points): 0–5 points, nutrition risk 0; 6–12 points, nutrition risk 1; 13–16 points, nutrition risk 2; 17–21 points, nutrition risk 3; 22–29 points, nutrition risk 4.

^a^ Sex and age differences lead to significant tumour‐type heterogeneity. Disease characteristics are the decisive factor for death risk in cancer patients. Moreover, there were no significant differences in most nutritional risk groups between adults (ages 18–64 years) and older patients (ages ≥ 65 years) and between males and females adjusting for specific tumour types (e.g., in patients with gastric cancer; Figure S2.2). To avoid a significant bias regarding cancer‐type heterogeneity, we did not include sex and age in the NWSS. This might be the reason why previous studies likewise did not include age and sex in their diagnostic tools for cancer cachexia.

^b^ Albumin levels were classified according to conventional clinical classifications and cut‐off values.

^c^ Albumin/globulin ratio (AGR) levels were classified according to conventional clinical classifications and cut‐off values.

^d^ Muscle loss is a key problem in cachexia. However, accurate body composition analysis is expensive and inconvenient in nutrition screening jobs. Therefore, many researchers recommend easily available body mass index (BMI) as an alternative indicator for diagnosis of cachexia. For easy clinical use, we chose the Chinese routine cut‐off values for BMI classification in our study.

^e^ The neutrophil/lymphocyte ratio (NLR) can easily be obtained using routine blood tests and is a widely recommended indicator for systemic inflammation and cachexia. However, the NLR has no clinical routine cut‐off value and was determined according to Youden index values calculated using the receiver operating characteristic (ROC) curves.

^f^ There is no standard HGS range for cancer patients. Thus, HGS was summarized using simple quartiles by sex and age groups (adult males [aged 18–64 years], adult females, elderly males [ages ≥ 65 years] and elderly females). Patients with an HGS value greater than the median for their group scored 0 point (normal), those with an HGS value greater than the lower quartile and less than the median for their group scored 1 point (moderate lesion) and those with an HGS value less than the lower quartile scored 2 points (severe lesion).

^g^ The weight loss sub‐scale of the Patient‐Generated Subjective Global Assessment (PG‐SGA) has a good grading classification for weight loss according two dimensions, including absolute value and rate of weight loss, and is used, as shown in Table S1.1.

^h^ Intake reduction was assessed by patient's self‐evaluation report, as shown in Table S1.2.

^i^ The physical activity function (PAF), anorexia and fatigue domain in a quality‐of‐life instrument designed by the European Organization for Research and Treatment of Cancer (EORTC QLQ‐C30) was included. The calculation of the raw score of the PAF and fatigue domain is complex. Therefore, we devised a simplified PAF and fatigue scale according to the hazard ratios (HRs) of the raw scores for these questions using Cox proportional hazards models, as listed in Tables S1.3, S1.4 and S1.5. The simplified scales yielded consistent diagnostic value as compared with raw scores (Figure S2.1).

^j^ The physical activity function (PAF), anorexia and fatigue domain in a quality‐of‐life instrument designed by the European Organization for Research and Treatment of Cancer (EORTC QLQ‐C30) was included. The calculation of the raw score of the PAF and fatigue domain is complex. Therefore, we devised a simplified PAF and fatigue scale according to the hazard ratios (HRs) of the raw scores for these questions using Cox proportional hazards models, as listed in Tables S1.3, S1.4 and S1.5. The simplified scales yielded consistent diagnostic value as compared with raw scores (Figure S2.1).

^k^ The physical activity function (PAF), anorexia and fatigue domain in a quality‐of‐life instrument designed by the European Organization for Research and Treatment of Cancer (EORTC QLQ‐C30) was included. The calculation of the raw score of the PAF and fatigue domain is complex. Therefore, we devised a simplified PAF and fatigue scale according to the hazard ratios (HRs) of the raw scores for these questions using Cox proportional hazards models, as listed in Tables S1.3, S1.4 and S1.5. The simplified scales yielded consistent diagnostic value as compared with raw scores (Figure S2.1).

#### 2.6.3 Step 3: Establishment of the Disease‐Weighted Scoring Scale (DWSS)

We established a simplified disease‐weighted scoring scale (DWSS) based on categories of tumour types and tumour burden statuses for overall patients.

##### 2.6.3.1 Tumour Types

Overall, 14 tumour types were classified into sequence variables, and Cox proportional hazards analysis was conducted using breast cancer (with the lowest death risk) as the reference. Tumour types with similar HRs and survivals were merged into four categories (weighted score): tumour type Category A (0; with the lowest death risk), including breast and cervical cancer and nasopharyngeal and endometrial carcinoma; tumour type Category B (2), including prostate, ovarian, bladder and colorectal cancer; tumour type Category C (4), including gastric, oesophageal, liver and lung cancer; and tumour type Category D (6; with the highest death risk), including pancreatic cancer and cholangiocarcinoma.

##### 2.6.3.2 Tumour Burden Status

Chronic hyperinflammatory/catabolism and metabolic disorders induced by an existing tumour is a key pathogenetic factor of DRM and cachexia [2, 4, 12]. Thus, we further established a tumour burden status system based on TNM stage and radical (existing tumours receiving scheduled radical anti‐tumour treatments or resected tumours receiving adjunct anti‐tumour treatments, without long‐term tumour burden) or non‐radical tumours (tumours receiving palliative anti‐tumour treatments or care, with long‐term tumour burden) to evaluate patients' long‐term tumour burden status.

Cachexia is mostly found in patients with advanced stage disease. Nevertheless, we found that many preoperative patients with early‐stage tumours also had low BMI, weight loss, sarcopenia and hyperinflammatory/catabolism and metabolic disorders induced by the unresected tumours but survived well and benefited from nutritional therapy, suggesting that clinical risk stratification including early‐stage tumours is needed for optimal classification and treatment. Thus, in this study, we included patients with radical Stage I–II tumours. Further, we found that in patients with stage IV tumours, patients with vital organ (i.e., lung, live and brain) metastasis or ≥ 2 distal organ metastases had significantly higher death risk than patients with ≤ 1 distal organ metastasis and without vital organ metastasis. Hence, we created eight groups according to TNM stage and radical or non‐radical tumour combinations and performed Cox proportional hazards analysis using patients with Stage I cancer (all radical tumours) as reference. The groups with similar HRs and survivals were combined together into five categories (weighted score): tumour burden A (0), radical Stage I–II tumours (36 patients with non‐radical Stage II tumours were excluded to avoid statistical bias); tumour burden B (1), radical Stage III tumours; tumour burden C (3), including non‐radical Stage III tumours and radical Stage IV tumours with ≤ 1 distal metastasis; tumour burden D (4), including non‐radical Stage IV tumours with ≤ 1 distal metastasis and radical Stage IV tumours with vital organ metastasis or ≥ 2 organ metastases; and tumour burden E (5), non‐radical Stage IV tumours with vital organ metastasis or ≥ 2 organ metastases.

Finally, the DWSS was established using a simple summation method (Table 3), which can serve as a complete alternative to complicated matrix methodology (Figure S2.3). The effects of specific disease characteristics on cachexia risk are illustrated in footnotes of Table 3.

**TABLE 3:** The disease‐weighted scoring scale (DWSS).

Tumour type^a^, ^b^, ^c^	Breast and cervical cancer and nasopharyngeal and endometrial carcinoma	0 point
	Bladder, ovarian, colorectal and prostate cancers	2 points
	Oesophageal, gastric liver and lung cancers	4 points
	Pancreatic cancer and cholangiocarcinoma	6 points
Tumour burden status^d^, ^e^	Radical Stage I–II tumour (patients with unresected but resectable tumour receiving scheduled radical surgery or radiotherapy, or resected tumour receiving adjuvant anti‐tumour treatments)	0 point
	Radical Stage III tumour	1 point
	Non‐radical Stage III tumour (patients with non‐radical tumour receiving palliative anti‐tumour treatments or palliative care); radical Stage IV tumour with ≤ 1 distal metastasis	3 points
	Non‐radical Stage IV tumour with ≤ 1 distal metastasis; radical Stage IV tumour with vital organ metastases or ≥ 2 organ metastasis	4 points
	Non‐radical Stage IV tumour with vital organ metastases or ≥ 2 organ metastasis	5 points

*Note:* The classification of the DWSS (0–11 points): disease risk I, 0–2 points; disease risk II, 3–4 points; disease risk III, 5 points; disease risk IV, 6–8 points; disease risk V, 9–11 points.

^a^ There were significant differences in death risk associated with different tumour types and tumour burden statuses. These differences may be related to differences in disease characteristics. Most tumour types of tumour type A and B categories are non‐vital‐organ and non‐gastrointestinal tumours (breast, cervical, bladder, prostate, ovarian cancer and endometrial carcinoma) and showed lower inflammatory burden/catabolism and metabolic disorders, less negative effects on digestive function, higher basic body mass index (BMI) levels and lower risk of death. These cancers have very low risk of severe malnutrition and death before occurrence of vital organ metastases. For example, a bone metastasis or pelvic adjacent organ metastasis may significantly reduce the quality of life due to related complications but would not be life threatening. However, once metastasis to a vital organ occurs, the risk of nutritional deterioration and death increases dramatically. Hence, patients with non‐radical vital organ metastases might have high risk of cachexia, despite a low overall risk in patients with these tumour types. Nasopharyngeal carcinoma and its associated anti‐tumour treatments (e.g., surgery and radical radiotherapy) significantly affect patients' oral feeding. However, patients with nasopharyngeal carcinoma maintain complete gastrointestinal digestion and absorption function by replacing oral feeding by infusion of a homogenate meal through a gastrostomy tube at home. Thus, patients with nasopharyngeal carcinoma showed a very low risk of malnutrition and death, as did patients with breast cancer. Food is almost completely digested and absorbed before it enters the colon; hence, digestive tract reconstruction in colorectal cancer after surgical resection has less effect on digestive function. Even in patients with non‐radical tumours, a simple fistulation can solve the problem of defecation while sparing eating and digestive function. Thus, patients with colorectal cancer also showed a low risk of malnutrition and death. Type C and D tumours are vital organ (lung and live cancer) or upper gastrointestinal tumours (gastric, oesophageal, liver and pancreatic cancers, and cholangiocarcinoma). Patients with these tumours had a high risk of death and nutritional deterioration, including higher inflammatory burden/catabolism and metabolic disorders, greater negative effects on digestive function, and lower basic BMI levels. Patients with radical Stage III upper gastrointestinal tumours also revealed risk of nutritional deterioration due to substantial negative effects on feeding and digestive function caused by the complicated post‐operative gastrointestinal reconstruction, which comprises removal of most or all of the stomach. After reconstruction, the function of cardia and/or pylorus is lost, and the food directly enters the jejunum. However, the tolerance of the jejunum to food volume, food category and density of energy and protein are significantly inferior to that of the stomach. In addition, the digestive enzyme secretion function is damaged, especially in patients with pancreatic cancer and cholangiocarcinoma. These negative effects lead to significant intake reduction, digestive and absorptive dysfunction and inevitable weight loss and nutritional deterioration. Pancreatic cancer and cholangiocarcinoma are rarely detected in the early stages (Stages I–II). In addition, patients with advanced pancreatic cancer and cholangiocarcinoma have very low chance of surgical resection and great negative effect on digestive function due to both preoperative gastrointestinal occupancy and complicated post‐operative gastrointestinal reconstruction. Hence, these patients have very high risk of death and nutritional deterioration. Notably, patients with non‐radical lung cancer may reveal dramatically increased energy wasting and nutritional reserve loss beyond what is expected based on food intake reduction, due to their high levels of hyperinflammation/catabolism and metabolic disorders and special complications (e.g., respiratory tract obstruction and pleural effusion), which further lead to increased breathing burden and energy wasting.

^b^ Few patients (*n* = 42, 0.04%) had multiple primary tumour types, and their death risk was mainly determined by the tumour types with the higher death risk. Therefore, the final score in patients with multiple primary tumour types should be determined by the highest total DWSS scores, and the extra scores of the coexisting tumour types need not be added.

^c^ All comorbidities, including chronic obstructive pulmonary disease (COPD), stroke, active tuberculosis, hypertension and diabetes, were excluded by multivariate Cox proportional hazards model. Hypertension and diabetes may be excluded because of their lower effect on death risk compared with that of tumours. However, COPD, stroke and active tuberculosis had great effects on death risk, especially patients with abortive tuberculosis, who all died within 25 months. They should be excluded because of the following reasons. Tuberculosis and COPD could cause extreme cachexia phenotype. However, in patients with cancer and with these comorbidities, the high risk of death may be attributed to the limitation to anti‐tumour treatments implementation, rather than to an independent cachexia phenotype unrelated to cancer cachexia. Vast evidence confirms that the potential risks of anti‐tumour treatments are likely to outweigh benefits in patients with these comorbidities. Our data showed that most of the patients with these comorbidities lost opportunities of radical anti‐tumour treatments and received palliative anti‐tumour treatments and care. This might be the reason why these high‐risk comorbidities were excluded by the multivariate model, adjusting for strategies of scheduled anti‐tumour treatments, because the decisive aetiological factors determining the risk of cachexia and death were radical and non‐radical tumours, and the clinicians had taken these factors into consideration when choosing a comprehensive strategy of anti‐tumour treatments.

^d^ Patients with non‐radical vital organ metastasis (liver, lung and brain) had higher death risk than patients with two or more non‐radical non‐vital‐organ metastases. Therefore, non‐radical vital organ metastasis should be assigned the highest tumour burden status score, regardless of the numbers of metastases.

^e^ It is generally believed that patients with recurrent tumours have higher death risk than those with primary tumours. However, our data showed that patients' death risk was mainly dependent on the actual tumour burden status rather than on the tumour recurrence. Patients with recurrent tumours had slightly higher death risk than those with primary tumours and the same tumour burden scores but demonstrated significantly better survival than patients with primary tumours and higher tumour burden scores. Furthermore, the recurrence status was excluded from the multivariate Cox models after adjusting for tumour burden status. Therefore, tumour recurrence was excluded.

##### 2.6.3.3 DWSS Classification (Disease Risk)

We determined a five‐grade classification of the DWSS (0–11 points) using the same method as described for the NWSS (Figure 2). Supporting Information S2 explains the process of establishing the DWSS.

#### 2.6.4 Step 4: Establishment of the Lumped Scale and Categories of Comprehensive Cachexia Risk

**FIGURE 3:**
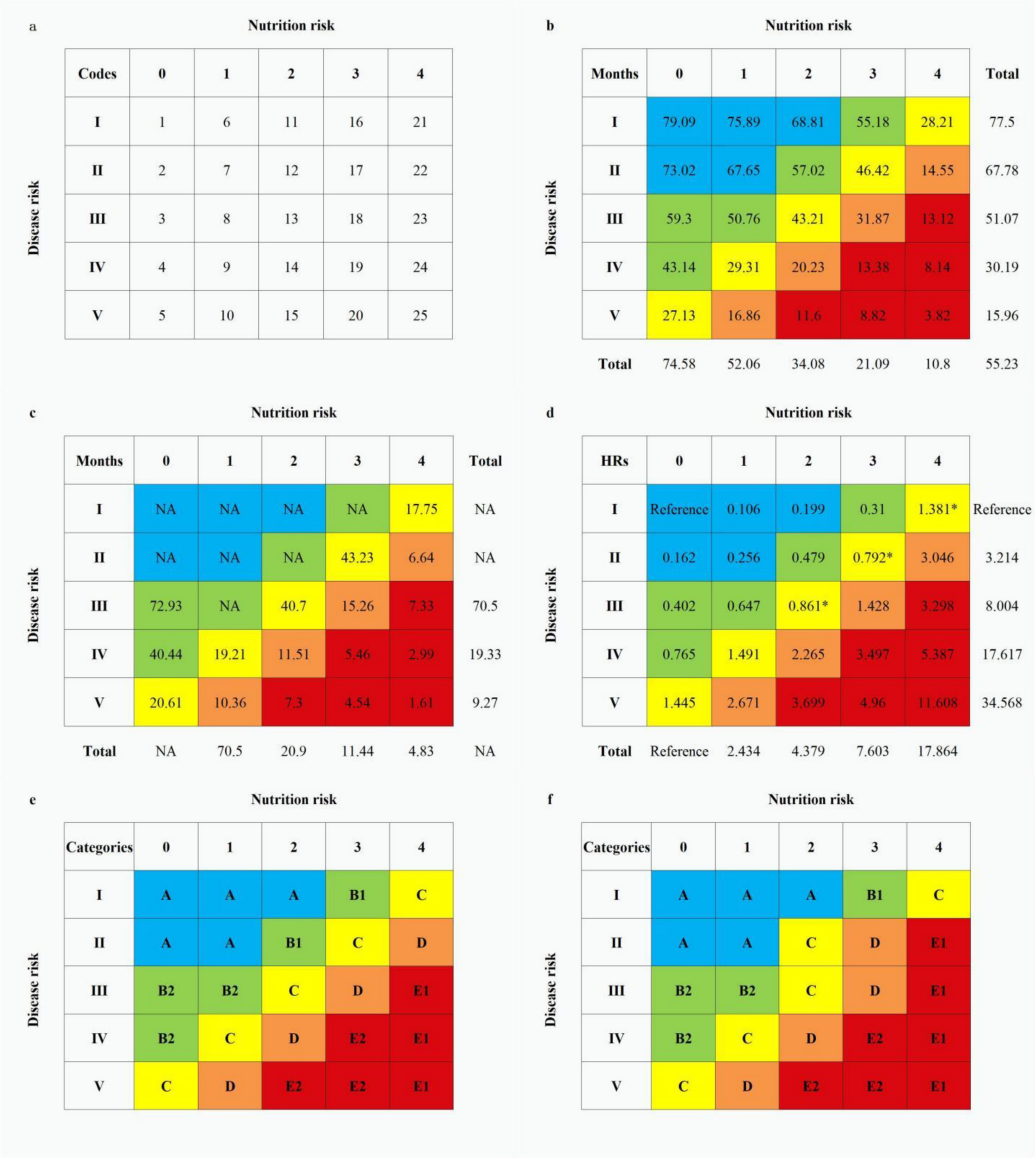
The 5 × 5 matrix of the lumped scale. *Notes:* Panels a to f represent a 5 × 5 matrix analysis of the five‐grade classification of nutrition and disease risk developed herein, for a total of 25 possible combinations. The (a) codes, (b) mean survivals (months), (c) median survivals (months), (d) estimated hazard ratios (HRs), (e) categories of comprehensive cachexia risks for overall patients and (f) the adjusted tumour‐type‐specific diagnoses for patients with tumour type Category A cancers, including breast and cervical cancer and nasopharyngeal and endometrial carcinoma. Reference categories represent the lowest nutrition and disease risks. NA, not applicable.

We further established a lumped scale (5 × 5 matrix) using classifications of both nutrition and disease risks (Figure 3) [17] and determined a five‐grade comprehensive cachexia risk category based on a combined evaluation of nutrition and disease risks, death risks and response to nutrition therapy and anti‐tumour treatments.

#### 2.6.5 Step 5: Validation

A validation set established the diagnostic scales and classifications according to the methods of the derivation set, and the time‐dependent ROC curves assessed the discrimination of the scales in predicting survival.

## 3 Results

### 3.1 Patient Characteristics

There were 10 022 and 2629 patients in the derivation set and validation set, respectively. Table 1 summarizes the patient characteristics.

### 3.2 The NWSS and Nutrition Risk

Table 2 presents the NWSS interpretation. Finer categorization of malnutrition elements in the five‐grade nutrition risk is as follows (Figure S2.1):

1 Malnutrition without hyperinflammatory/catabolism and metabolic disorders

- Nutrition risk 0: No or mild malnutrition with no or low nutritional/functional reserve impairment (e.g., low BMI, fatigue and HGS and PAF decline) and/or indicators of ongoing nutritional deterioration (e.g., food intake reduction and weight loss).
- Nutrition risk 1: Moderate or severe malnutrition with increased nutritional/functional reserve impairment and ongoing nutritional deterioration, but with no or low‐level hyperinflammatory/catabolism and metabolic disorders, including anorexia, increased NLR and low albumin and AGR levels.

2 Malnutrition with hyperinflammatory/catabolism and metabolic disorders

- Nutrition risk 2: Severe malnutrition with added transient (in preoperative patients) or chronic (in patients with non‐radical tumours) hyperinflammatory/catabolism and metabolic disorders.
- Nutrition risk 3: Severe malnutrition with prevailing transient or chronic hyperinflammatory/catabolism and metabolic disorders.
- Nutrition risk 4: Severe‐extreme derangement of global nutritional status.

The HRs (95% CIs) for death and mean (median) months for survival for the nutrition risk of 0, 1, 2, 3 and 4 were reference, 2.434 (2.231/2.654), 4.379 (3.934/4.874), 7.603 (6.78/8.527) and 17.864 (15.249/20.927), respectively (*p* < 0.001), and 68.06 (NA), 52.71 (79.69), 38.4 (21.96), 25.09 (10.8), and 8.84 (2.99) months, respectively (*p* < 0.001).

### 3.3 The DWSS and Disease Risk

Table 3 presents DWSS interpretation. Using matrix methodology, the characteristics of the five‐grade disease risk are shown in Figure S2.3 using a matrix methodology as follows:

- Disease risk I: Patients with tumour type A and tumour burden A–B or tumour type B and tumour burden A.
- Disease risk II: Patients with tumour type A and tumour burden C–D, patients with tumour type B and tumour burden B or patients with tumour type C and tumour burden A.
- Disease risk III: Patients with tumour type A and tumour burden E, patients with tumour type B and tumour burden C or patients with tumour type C and tumour burden B.
- Disease risk IV: Patients with tumour type B and tumour burden D–E, patients with tumour type C and tumour burden C–D or patients with tumour type D and tumour burden A–B.
- Disease risk V: Patients with tumour type C and tumour burden E or patients with tumour type D and tumour burden C, D or E.

The HRs for death and mean (median) months survival for disease risk I, II, III, IV and V categories were reference, 3.214 (2.696/3.832), 8.004 (6.74/9.505), 17.617 (15.02/20.662), and 34.568 (29.152/40.991), respectively (*p* < 0.001), and 77.5 (NA), 67.78 (NA), 51.07 (70.5), 30.19 (19.33) and 15.96 (9.27) months, respectively (*p* < 0.001).

### 3.4 The Lumped Scale and Comprehensive cachexia Risk

The lumped scale (5 × 5 matrix) determined a five‐graded comprehensive cachexia risk (Figure 3) as follows:

- Comprehensive cachexia risk A: No cachexia risk, patients with the lowest disease and nutrition risks have no cachexia risk.
- Comprehensive cachexia risk B: comprehensive cachexia risk, patients with a potential cachexia risk with hyperinflammatory/catabolism and metabolic disorders (nutrition risk 2–3), which needs to be re‐evaluated after radical anti‐tumour treatments (B1), or patients without hyperinflammatory/catabolism and metabolic disorders (nutrition risk 0–1), but with high risk for cachexia due to high disease risk (e.g., irreversible gastrointestinal dysfunction caused by non‐radical tumour occupy and/or complicated post‐operative digestive reconstruction in patients with digestive tumours and/or vital organ dysfunction in patients with non‐radical vital organ tumours and/or metastases) (B2).
- Comprehensive cachexia risk C: Comprehensive pre‐cachexia, a stage before comprehensive cachexia with drastically increased death risk; progression of cachexia may vary, depending on a high nutrition risk or high disease risk.
- Comprehensive cachexia risk D: Comprehensive cachexia, patients with high death risk and fewer benefits from NT due to both high disease and nutrition risks.
- Comprehensive cachexia risk E: Comprehensive refractory cachexia, patients with very high death risk and clinically refractory to nutrition therapy and anti‐tumour treatments due to extreme nutritional deterioration (E1) and/or the highest disease risk (E2).

The HRs (95% CIs) for death and mean (median) months survival for the comprehensive cachexia risk A, B, C, D and E were reference, 4.517 (4.033/5.058), identification of actual 9.755 (8.73/10.901), 16.901 (14.995/19.049) and 31.879 (28.244/35.981), respectively (*p* < 0.001), and 74.58 (NA), 52.22 (70.5), 34.45 (21.21), 21.21 (11.61) and 10.79 (4.83) months, respectively (*p* < 0.001).

### 3.5 Responses to Nutrition Therapy and Anti‐tumour Treatments Differed by Nutrition and Comprehensive cachexia Risks

Patients receiving nutrition therapy had significantly worse nutrition status and survival than those without nutrition therapy, due to clinical selection bias. In patients receiving pre‐hospitalization nutrition therapy, hospitalization nutrition therapy and not receiving nutrition therapy, the median scores (interquartile range) of the NWSS were 11 (7/16), 7 (3/12) and 6 (3/9) points, respectively (*p* < 0.001); and the mean survival were 54.19, 44.40 and 56.57 months, respectively (*p* < 0.001).

**FIGURE 4:**
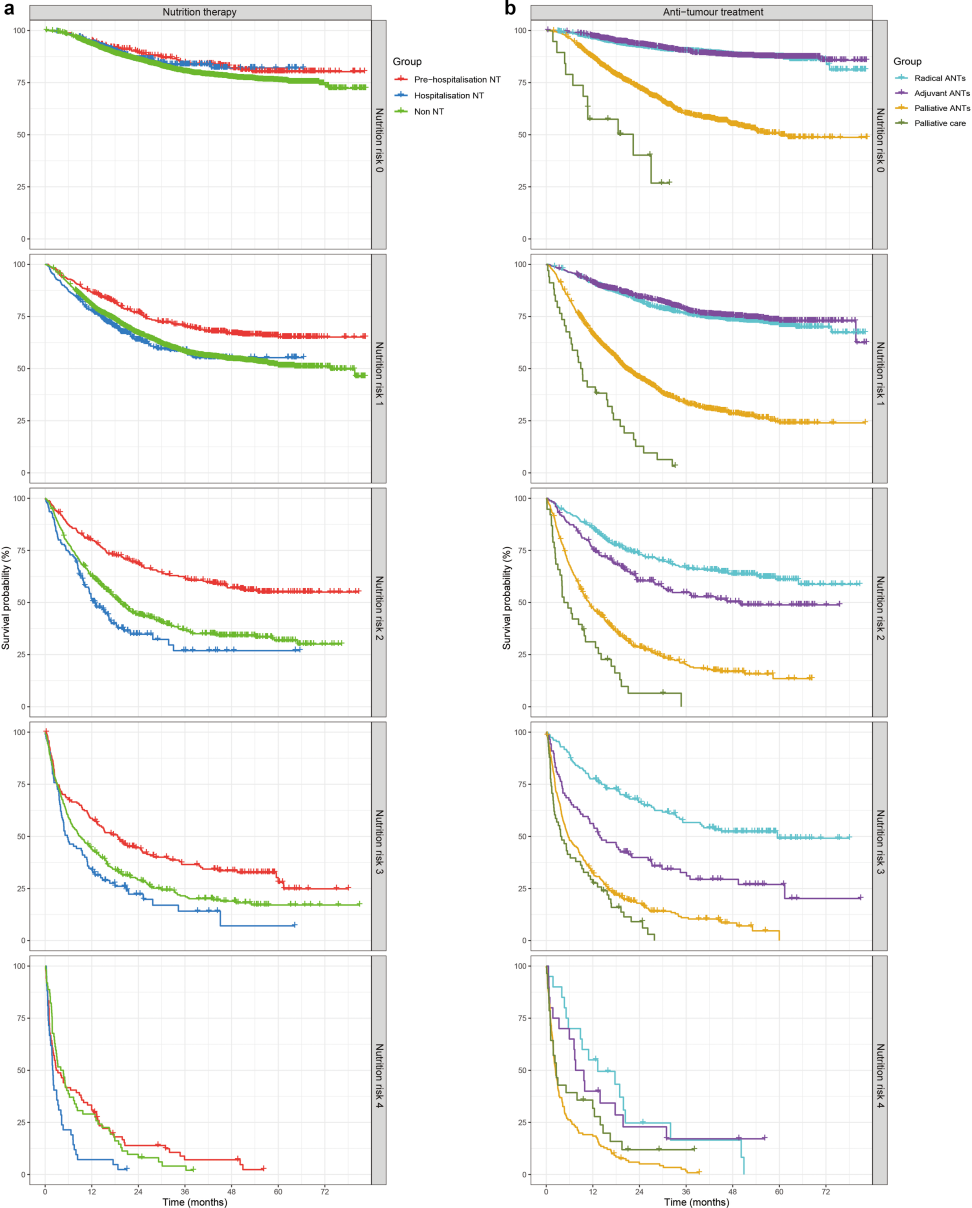
Survival in nutrition therapy (NT) and anti‐tumour treatments (ANTs) subgroups in regard to nutrition risk. Notes: (a) There were no differences in survival among NT subgroups given a nutrition risk 0 of the nutrition‐weighted scoring scale (NWSS); in nutrition risk 1–3 classification, patients receiving pre‐hospitalization NT had the best survivals, whereas hospitalization NT did not guarantee benefits; in nutrition risk 4 classification, patients did not benefit from NT. Pre‐hospitalization NT (receiving NT before hospitalization); hospitalization NT (receiving NT after hospitalization); and non NT (not receiving NT). P1, pre‐hospitalization NT vs. non NT; P2, hospitalization NT vs. non NT; P3, pre‐hospitalization NT vs. hospitalization NT. (b) In nutrition risk 0–3 classifications, patients benefited from ANTs, with prolonged survivals as compared with those receiving palliative care; in nutrition risk 4 classification, patients benefited from radical ANTs. Radical ANTs (radical surgery and radiotherapy), adjuvant ANTs (adjuvant chemotherapy, radiotherapy, molecular targeted therapy, endocrine therapy after radical surgery), palliative ANTs (palliative surgery, chemotherapy, radiotherapy, molecular targeted therapy and immunotherapy in patients with non‐radical tumours) and palliative care (symptomatic support treatments administered only for relieving pain and improving quality of life). P1, radical ANTs vs. adjuvant ANTs; P2 radical ANTs vs. palliative ANTs; P3, radical ANTs vs. palliative care; P4 adjuvant ANTs vs. palliative ANTs; P5 adjuvant ANTs vs. palliative care; P6 palliative ANTs vs. palliative care.

**FIGURE 5:**
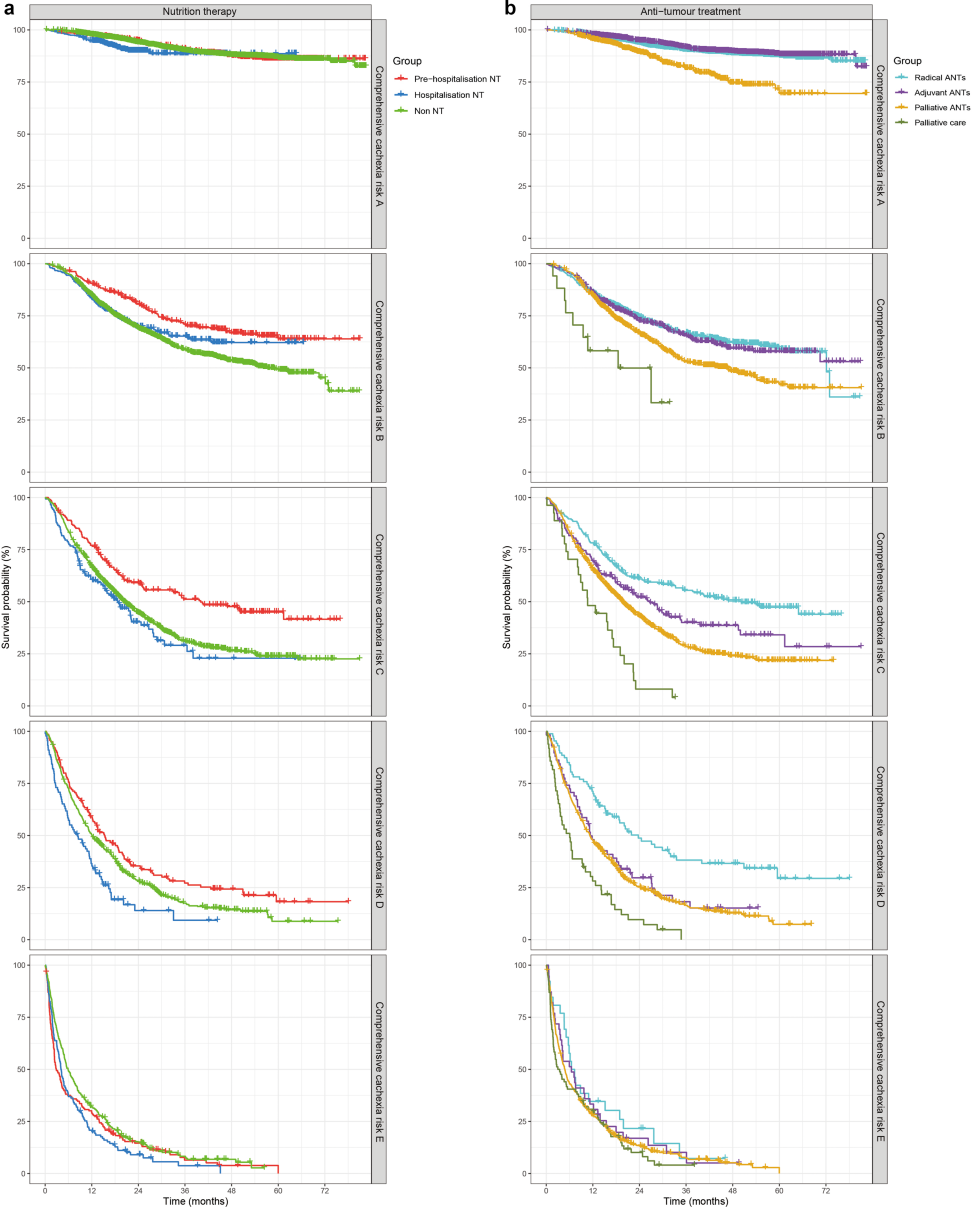
Survival in nutrition therapy (NT) and anti‐tumour treatments (ANTs) subgroups in regard to comprehensive cachexia risk. Notes: (a) There were no differences in survival among NT subgroups in regard to comprehensive cachexia risk A of the lumped scale. In comprehensive cachexia risk B–D category, patients receiving pre‐hospitalization NT had the best survivals, whereas hospitalization NT did not guarantee benefits. In comprehensive cachexia risk E category, patients did not benefit from NT. Pre‐hospitalization NT (receiving NT before hospitalization); hospitalization NT (receiving NT after hospitalization); and non NT (not receiving NT). P1, pre‐hospitalization NT vs. non NT; P2, hospitalization NT vs. non NT; P3, pre‐hospitalization NT vs. hospitalization NT. (b) In comprehensive cachexia risk A–D category, patients benefited from ANTs, demonstrating prolonged survivals compared with those receiving palliative care. In comprehensive cachexia risk E category, patients did not benefit from ANTs. Radical ANTs (radical surgery and radiotherapy), adjuvant ANTs (adjuvant chemotherapy, radiotherapy, molecular targeted therapy and endocrine therapy after radical surgery), palliative ANTs (palliative surgery, chemotherapy, radiotherapy, molecular targeted therapy and immunotherapy in patients with non‐radical tumours) and palliative care (symptomatic support treatments only for relieving pain and improving quality of life). P1, radical ANTs vs. adjuvant ANTs; P2 radical ANTs vs. palliative ANTs; P3, radical ANTs vs. palliative care; P4 adjuvant ANTs vs. palliative ANTs; P5 adjuvant ANTs vs. palliative care; P6 palliative ANTs vs. palliative care.

However, comprehensive analyses including nutrition risk and comprehensive cachexia risk corrected these biases and allowed the identification of actual beneficial effects of nutrition therapy. In nutrition risk 0 and comprehensive cachexia risk A groups, there were no significant differences in survival among nutrition therapy subgroups, suggesting that the role of nutrition therapy is more related to the prevention of initial nutritional deterioration without significant impact on long‐term survival in these groups. In nutrition risk 1–3 and comprehensive cachexia risk B–D groups, patients significantly benefited from pre‐hospitalization nutrition therapy with the best survivals among nutrition therapy subgroups. Compared with patients without nutrition therapy, patients receiving hospitalization nutrition therapy did not reveal prolonged survivals in nutrition risk 1 and comprehensive cachexia risk B–C groups but revealed significantly worse survivals in nutrition risk 2–3 and comprehensive cachexia risk D groups. Patients with nutrition risk 4 did not benefit from pre‐hospitalization nutrition therapy but still benefited from radical anti‐tumour treatments compared with patients receiving palliative care (medium [medians] survival, 19.00 [13.25] vs. 7.39 [2.56] months [*p* = 0.015]). Patients with comprehensive cachexia risk E did not benefit from either pre‐hospitalization nutrition therapy or anti‐tumour treatments in terms of survival (Figures 4 and 5).

### 3.6 Validation Set

In the time‐dependent ROC curves, AUCs for the NWSS, DWSS and lumped scale revealed that mortality risk in the validation set reached ≥ 90% of that in the derivation set for the corresponding scales at all time nodes (Figure S5.3).

## 4 Discussion

In this study, we aimed to provide comprehensive evaluation scales for cachexia in patients with solid tumours by combining multidimensional disease and nutritional parameters and outcomes.

Several previous proposed diagnostic criteria for cachexia, based on sarcopenia and BMI‐adjusted‐weight loss only [2, 15, 16, 17], could not distinguish pre‐cachexia from non‐cachexia [18, 19]. Reportedly, this could be addressed by including indicators of inflammatory burden/catabolism and metabolic disorders [27, 28]. Hence, we first established an NWSS using multidimensional nutritional parameters and determined a grading classification of nutrition risk, as previously reported. Our results showed that a malnutrition without hyperinflammatory/catabolism and metabolic disorders (nutrition risk 0–1), which might pertain to non‐cachexia, was the major nutritional problem in patients with cancers, including patients with advanced tumours; but a malnutrition with hyperinflammatory/catabolism and metabolic disorders (nutrition risk 2–3), which might pertain to cachexia categories [12], occupied less proportion of nutritional problem.

Notably, the association between nutrition risk and outcome was also influenced by disease characteristics and disease‐associated risk. Moreover, whereas the grade of disease risk may primarily reflect the clinical impact of disease burden per se, disease burden is also a major driving factor for the deterioration of nutritional status [26]. Patients with higher disease risk had lower BMI, more pronounced food intake reduction and weight loss and functional deterioration with fatigue and PAF and HGS decline.

Association between disease characteristics and hyperinflammatory/catabolism and metabolic disorders markers was more intriguing. Patients with resectable but unresected tumours receiving scheduled surgery or those with non‐radical tumours receiving palliative anti‐tumour treatments or care had a higher prevalence of increased NLR and low albumin compared with those with resected tumours receiving adjunct anti‐tumour treatments. These observations suggest that the existing tumour plays a major role in the onset of hyperinflammatory/catabolism and metabolic disorders and might cause a high but transient nutrition risk in preoperative patients, which may be reduced after tumour resection (Figure S3.1) [29]. Patients with radical tumours receiving radical or adjuvant anti‐tumour treatments might have no or low long‐term tumour burden and associated chronic hyperinflammatory/catabolism and metabolic disorders, whereas patients with non‐radical tumours have a long‐term tumour burden and therefore high risk of hyperinflammatory/catabolism and metabolic disorders. This significantly influenced the disease duration and burden and diagnosis of cachexia.

Therefore, we further established a lumped scale based on both nutrition and disease risks, allowing for a comprehensive assessment of cachexia risk. For instance, significant tumour burden heterogeneity was observed in the disease risk II–IV subgroups. Patients with higher‐risk tumour types and radical tumours and those with lower‐risk tumour types and non‐radical tumours coexist in the same subgroup; this may mainly reflect differences in death risk in different tumour types. Furthermore, when considering nutrition risks, tumour burden heterogeneity had a limited impact on death risk and benefits from nutrition therapy in nutrition risk 0–1, but it was associated with substantial differences in death risk and benefits from nutrition therapy in the nutrition risk 2–4 groups. Patients with radical tumours showed a low increase in death risk and benefited from pre‐hospitalization nutrition therapy, suggesting that a malnutrition with potentially transient hyperinflammatory/catabolism and metabolic disorders in preoperative patients is a potential cachexia risk requiring re‐evaluation after surgery. Conversely, patients with non‐radical tumours had dramatically increased death risk with increased nutrition risk, and those receiving pre‐hospitalization nutrition therapy had worse survivals than patients without nutrition therapy, suggesting that a malnutrition with chronic hyperinflammatory/catabolism and metabolic disorders in patients with non‐radical tumours establishes the presence of cachexia (Figure S4.1).

In the disease risk V subgroup, wherein all patients had non‐radical tumours, those receiving pre‐hospitalization nutrition therapy demonstrated worse survival, even in the presence of low nutrition risk 0–1. In these patients, the highest disease risk (e.g. irreversible gastrointestinal dysfunction caused by the non‐radical digestive tumour and/or vital organ dysfunction caused by non‐radical vital organ tumours and/or metastases) might drive a strong nutritional deterioration risk, poor response to nutrition therapy, and death, despite no hyperinflammatory/catabolism and metabolic disorders.

These results support the concept that the risk of cachexia progression depends on combined nutritional deterioration and disease burden, and cachexia (‘bad condition’, e.g., very high death risk and clinically refractory to nutrition therapy and anti‐tumour treatments) can be induced by a very advanced cancer (pre‐terminal) or rapidly progressive tumour [2]. Thus, we suggest that definitions of cachexia focusing only on nutritional parameters might prevent comprehensive stratification and assessment of clinical risk. Therefore, we proposed a novel definition and grading diagnosis of comprehensive cachexia risk based on comprehensive consideration of disease characteristics, nutrition risk and outcomes. The comprehensive cachexia risk is defined as a potentially irreversible and progressing multifactorial clinical syndrome driven by both disease progression and multidimensional nutritional/functional deterioration, which eventually becomes refractory to nutrition therapy and anti‐tumour treatments and causes death. It may be difficult to determine the sequence of disease progression and nutritional/functional deterioration in many patients; however, cachexia, which is a continued wasting syndrome until extreme emaciation and weakness, is inevitably caused by chronic burdens driven by incurable diseases [2, 4]. Thus, disease characteristics may be the basic aetiological factors and basis of differential diagnosis for comprehensive cachexia.

As a high disease risk might become a major determinant of cachexia, we classified many patients with high disease risks but low nutrition risks as comprehensive cachexia risk B–D (comprehensive cachexia risk, pre‐cachexia and cachexia); monitoring and supplemental diagnosis for cachexia are needed to enhance clinical awareness of the high risk of nutritional deterioration. A refined qualitative diagnosis of comprehensive cachexia risk B1 (a potential cachexia risk needs to be reevaluated after radical anti‐tumour treatments) and B2 (patients with low nutrition risk, but with high risk to develop cachexia due to high disease risk) might be clinically useful to rank different pro‐cachectic mechanisms and clinical risks to guide early diagnosis and individualized treatment [1]. Due to limited data availability, we did not provide refined qualitative diagnosis within comprehensive cachexia risk categories C and D.

Moreover, patients with low disease risks were more tolerant to nutritional deterioration with less increased death risk along with elevated nutritional risks, whereas patients with high disease risks were prone to nutritional deterioration with drastically increased death risk along with elevated nutritional risk, suggesting that using a common single nutrition risk cut‐off value may not be appropriate for all patients with different disease risks. Our data showed that patients with disease risk I–II had a very low death risk regardless of cachexia or not as per the Consensus 2011 (Figure S4.2). Thus, we determined developed a novel graded comprehensive cachexia risk classification model based on multiple cut‐off points of nutrition and disease risks, exhibiting differences in risk and developing trajectory of cachexia in patients with different tumour types and tumour burdens and providing an operable tumour‐type‐specific diagnosis of comprehensive cachexia risk (Figure S5.1).

Patients with tumour type A (breast and cervical cancer and nasopharyngeal and endometrial carcinoma) and B cancers (bladder, ovarian, colorectal and prostate cancers) had lower hyperinflammatory/catabolism and metabolic disorders, less negative effects on digestive function and lower nutrition risk; they were also more tolerant to nutritional deterioration with less increased death risk. Fewer of them were diagnosed as having nutrition risk 2–4 and comprehensive cachexia risks C–E. However, all patients with non‐radical vital organ metastases might have a high risk of cachexia. A necessary correction diagnosis for patients with type A tumours should be noted, as those in codes 12, 17 and 22 of the lumped scale should be diagnosed with comprehensive pre‐cachexia, cachexia and refractory cachexia, respectively (Figure 3). Patients with tumour type C (oesophageal, gastric liver and lung cancers) and D (pancreatic cancer and cholangiocarcinoma) had higher hyperinflammatory/catabolism and metabolic disorders, greater negative effects on digestive function caused by preoperative tumour occupancy and complicated post‐operative gastrointestinal reconstruction and higher nutrition risks; they were also more prone to nutritional deterioration with drastically increased death risk. Therefore, more patients in these groups were diagnosed as having comprehensive cachexia risks C–E. Moreover, all patients with pancreatic cancer and cholangiocarcinoma (tumour type D) should be considered as having comprehensive cachexia risk, with the lowest graded diagnosis being comprehensive cachexia risk B2 (in code 4), as patients with pancreatic cancer and cholangiocarcinoma have dramatic digestive dysfunction, low opportunity of radical resection and poor response to multiple anti‐tumour treatments [30].

Last but not least, the novel graded comprehensive cachexia risk model based on multiple cut‐off points of nutrition risks in different disease risks more effectively evaluates patients' responses to nutrition therapy and anti‐tumour treatments, compared with the Consensus 2011 criteria (Figure S4.3). In the context of comprehensive cachexia risk B (cachexia risk) and C (pre‐cachexia), patients significantly benefited from pre‐hospitalization nutrition therapy (a pre‐emptive nutritional pre‐rehabilitation) with prolonged survivals, whereas patients receiving hospitalization nutrition therapy did not reveal prolonged survivals, compared with those without nutrition therapy. In comprehensive cachexia risk D (cachexia) category, patients still benefited from pre‐hospitalization nutrition therapy with less prolonged survival, whereas patients receiving hospitalization nutrition therapy had worse survivals, compared with those without nutrition therapy, suggesting poor responses to nutrition therapy due to high disease risk and/or nutrition risk and urgent needs of strengthened nutrition therapy in this subgroup [20, 31]. Regarding comprehensive cachexia risk E (refractory cachexia), patients were clinically refractory to nutrition therapy and anti‐tumour treatments and had a median survival of 4.83 months. The definition of refractory cachexia is essentially clinical [2]; and the course of refractory cachexia might be not limited in 3 months when the unresponsiveness to anti‐tumour treatments is considered [2]. The clinically refractory to anti‐tumour treatments are not always caused by the nutritional/functional deterioration, but patients clinically refractory to anti‐tumour treatments and disease progression would inevitably lead to nutritional deterioration. Therefore, pre‐emptive attention should be paid to the risk of cachexia when a patient has non‐radical diseases, rather than after the occurrence of irreversible nutritional/functional deterioration.

### 4.1 Strengths and Limitations

Our study has several strengths. First, the NWSS highlights the underlying role of hyperinflammatory/catabolism and metabolic disorders in suboptimal benefits from nutrition therapy and increased death risk. Second, although a simplified scale with missing details, the DWSS allows evaluation of the impact of disease characteristics on comprehensive cachexia risk. Third, the lumped scale determines more sophisticated grading diagnoses of comprehensive cachexia risks based on multiple cut‐off values of nutrition risk factors associated with poor outcomes in different disease risk subgroups, which provide a tumour‐type‐specific diagnosis of cachexia. Fourth, our NWSS, DWSS and lumped scale yielded consistently good prognostic values in patients with different tumour types and tumour burden status (Figure S5.2) and in patients from the validation set (Figure S5.3), suggesting that they have large applicability, reproducibility and reliability. Nevertheless, a more refined tumour‐type‐specific scale for tumour‐type‐specific disease risk and comprehensive cachexia risk for each tumour type based on more detailed specific disease characteristics is warranted.

There are also some limitations. First, this was an observational study; thus, the results should be interpreted with caution. Second, there are significant differences in the prevalence of specific cancer types and body composition between Chinese and Western populations, which may prevent universal utilization of the current scales. However, we found that disease characteristics and multidimensional nutritional deterioration with or without hyperinflammatory/catabolism and metabolic disorders, rather than a low BMI or weight loss unrelated to disease factors, bear strongest clinical and prognostic value, and the lumped scale provided an operable tumour‐type‐specific diagnosis of comprehensive cachexia risk. Thus, the lumped scale may be potentially useful in Western populations; this needs to be validated through further research.

## 5 Conclusions

We systematically proposed a novel definition of comprehensive cachexia risk and its grading diagnostic criterion for patients with solid tumours based on disease characteristics, nutritional parameters and clinical outcomes. Our novel scales may further develop the current consensus towards a more specific and operable graded diagnosis of cancer cachexia. Our findings need to be validated through future studies.

## Ethics Statement

This study has been approved by the workgroup of the INSCOC and the human research ethics committees of all participating hospitals and has therefore been performed in accordance with the ethical standards laid down in the 1964 Declaration of Helsinki and its later amendments.

## Conflicts of Interest

The authors declare no conflicts of interest.

## Supporting information


**Table S1.1.** Weight loss scale scores of the Patient‐Generated Subjective Global Assessment (PG‐SGA).
**Table S1.2.** Quantitative assessment (weighted score) of food intake reduction.
**Table S1.3.** Refined questionnaire scale of physical activity function.
**Table S1.4.** Refined questionnaire scale for fatigue.
**Table S1.5.** Refined questionnaire scale for anorexia.
**Figure S1.1.** Receiver operator characteristic (ROC) curves for determining the sensitivity and specificity of the refined scales and the raw scores for detecting risk of death.
**Table S2.1.** Nutritional/functional and disease indicators related to death risk in the univariate Cox proportional hazards analysis.
**Table S2.2.** Nutrition and disease indicators related to death risk on multivariate Cox proportional hazards modelling.
**Table S2.3.** Establishment steps (hazard ratios [HRs] and survivals of each category) of the nutrition‐weighted scoring scale.
**Figure S2.1.** Proportion of items (malnutrition elements) in classifications of the nutrition‐weighted scoring scale (NWSS).
**Figure S2.2.** Survival in different subgroups defined according to sex and age in nutrition risk classifications of the nutrition‐weighted score scale.
**Table S2.4.** Establishment steps (hazard ratios [HRs] and survival for each category) for the disease‐weighted scoring scale.
**Figure S2.3.** Matrix of the disease‐weighted scoring scale (DWSS) combining both tumour type categories and tumour burden status.
**Figure S3.1.** Incidence of the indicators of hyperinflammatory/catabolism and metabolic disorders in different disease characteristics and nutritional status.
**Figure S4.1.** The tumour burden heterogeneity and its associated differential diagnosis of the comprehensive cancer cachexia risk.
**Figure S4.2** The survivals of patients with cachexia as per consensus 2011 or not among different tumour burden status and disease risk subgroups
**Figure S4.3** Survival in nutrition therapy (NT) and anti‐tumour treatments (ANTs) subgroups in regard to diagnosis as per the consensus 2011.
**Figure S5.1.** Comprehensive risk diagnosis of cachexia in patients with gastric cancer.
**Figure S5.2.** The new scales yielded consistent distinguishing value regarding a variety of disease characteristics.
**Figure S5.3.** Time‐dependent receiver operator characteristic curves for determining the sensitivity and specificity of the different scales for detecting mortality risk.

## Data Availability

Individual participant data that underlie the results reported in this article, though not available in a public repository, will be made available to other researchers upon reasonable request (i.e., to researchers who provide a methodologically sound proposal) after de‐identification. These data include databases, the study protocol, the statistical analysis plan and the analytic code underlying these findings. The data will be available beginning 3 months and ending 5 years following article publication. Proposals should be directed to huchunlei212@126.com; to gain access, data requestors will need to sign a data access agreement.
