# Supplementary material for: A Novel Definition and Grading Diagnostic Criteria for Tumour‐Type‐Specific Comprehensive Cachexia Risk
Source: J Cachexia Sarcopenia Muscle. 2025 Mar 21;16(2):e13744. doi: 10.1002/jcsm.13744 (PMC11926632; doi:10.1002/jcsm.13744)
Supplement: Supplementary file 1 — Table S1.1. Weight loss scale scores of the Patient‐Generated Subjective Global Assessment (PG‐SGA). Table S1.2. Quantitative assessment (weighted score) of food intake reduction. Table S1.3. Refined questionnaire scale of physical activity function. Table S1.4. Refined questionnaire scale for fatigue. Table S1.5. Refined questionnaire scale for anorexia. Figure S1.1. Receiver operator characteristic (ROC) curves for determining the sensitivity and specificity of the refined scales and the raw scores for detecting risk of death. Table S2.1. Nutritional/functional and disease indicators related to death risk in the univariate Cox proportional hazards analysis. Table S2.2. Nutrition and disease indicators related to death risk on multivariate Cox proportional hazards modelling. Table S2.3. Establishment steps (hazard ratios [HRs] and survivals of each category) of the nutrition‐weighted scoring scale. Figure S2.1. Proportion of items (malnutrition elements) in classifications of the nutrition‐weighted scoring scale (NWSS). Figure S2.2. Survival in different subgroups defined according to sex and age in nutrition risk classifications of the nutrition‐weighted score scale. Table S2.4. Establishment steps (hazard ratios [HRs] and survival for each category) for the disease‐weighted scoring scale. Figure S2.3. Matrix of the disease‐weighted scoring scale (DWSS) combining both tumour type categories and tumour burden status. Figure S3.1. Incidence of the indicators of hyperinflammatory/catabolism and metabolic disorders in different disease characteristics and nutritional status. Figure S4.1. The tumour burden heterogeneity and its associated differential diagnosis of the comprehensive cancer cachexia risk. Figure S4.2 The survivals of patients with cachexia as per consensus 2011 or not among different tumour burden status and disease risk subgroups Figure S4.3 Survival in nutrition therapy (NT) and anti‐tumour treatments (ANTs) subgroups in regard to diagnosis as per the [file JCSM-16-e13744-s001.docx]

**A Novel Definition and Grading Diagnostic Criteria for Tumour-type-specific Comprehensive Cachexia risk: Supplementary File**

**Section 1. Associated disease and nutrtion indicators**

**Part 1 *Determination of associated disease and nutrtion indicators***

The available disease and nutrtion indicators related to cachexia in the Investigation on Nutrition Status and its Clinical Outcome of Common Cancers (INSCOC) database were selected based on a thorough review of the previous literature.[4, 5, 7]

***Nnutritional/functional indicators***

The domain of the muscle mass and body composition had multiple available indicators. The body mass index (BMI), middle arm circumference (MAMC), appendicular skeletal muscle mass (ASM) and appendicular skeletal muscle index (ASMI) were calculated using anthropometric data with the following formula: BMI (kg/m^2^)=weight (kg)/height (m)^2^;[20] MAMC (cm)=middle arm circumference (MAC) (cm)−3.14×triceps skin fold [TSF] (mm)/10;[13] ASM (kg)=0.193×body weight (kg)+0.107×height (cm)−4.157×sex(male=1 and female=2)−0.037×age (year)−2.631;[19] and ASMI=ASM/height (m)^2^. The fat free mass (FFM) was derived from bioelectrical impedance analysis (BIA), and fat free mass index (FFMI) divided by height: FFMI (kg/m^2^)=FFM (kg)/height (m)^2^. Due to significant differences in body composition between males and females, percent ASMI (%ASMI), MAMC (%MAMC) and FFMI (%FFMI) adjusted for sex-specific reference values were calculated and used in further analyses as follows: measurement value/reference value×100%. References values were 25.3 and 23.2 cm for MAMC,[14] 7.0 and 5.4 kg/m^2^ for ASMI,[10] and 18.6 and 15.7 kg/m^2^ for FFMI,[15] in men and women respectively.

The weight loss sub-scale (Table 1.1) of the Patient-Generated Subjective Global Assessment (PG-SGA) includes a comprehensive grading classification for weight loss, with both absolute value and rate of weight loss.[16] 96.5% (n=9,674/10,022) of patients had available one-month weight loss. If one-month weight loss information was missing, the six-month weight loss was used. Food intake information and food intake reduction were obtained by patient's self-evaluation report, as shown in Table 1.2.

The domain of the physical activity function (PAF) also had multiple available indicators, including the Karnofsky score (KPS), the PAF subscale (P-PAF) of the PG-SGA, and the PAF (E-PAF) domain in a quality-of-life instrument designed by the European Organisation for Research and Treatment of Cancer (EORTC QLQ-C30).[3] The fatigue and anorexia domains in the EORTC QLQ-C30 were also included. These indicators were obtained according to each corresponding scales. The calculations of the raw score of the E-PAF, fatigue and anorexia domain of the EORTC QLQ-C30 is complex.[3] Therefore, we redefined new simplified scales according to hazard ratios (HRs) of the raw scores for these questions using Cox proportional hazards models (Table 1.3, 1.4 and 1.5). Simplified scales yielded consistent diagnostic value when compared with raw scores (Figure 1.1).

Non-dominant handgrip strength (HGS) was also measured. According to the Asian Working Group for Sarcopenia (AWGS) diagnostic consensus on sarcopenia in 2019, a diagnosis of sarcopenia was made in the presence of both low HGS (men<28 kg, women<18 kg) and low ASMI (men<7.0 kg/m^2^, women<5.4 kg/m^2^).[10]

The diagnosis of cachexia as per Consensus 2011 was ascertained following fulfilment of any of these criteria:[12]

- Weight loss>5% over the past six months (in the absence of simple starvation),
- Weight loss>2% in individuals already showing depletion according to their current BMI levels (BMI <20 kg/m²), or
- Weight loss>2% and skeletal muscle depletion. Skeletal muscle depletion was using MAMC (men<32 cm², women<18 cm²).

The inflammation and catabolic drivers, including plasma neutrophil/lymphocyte ratio (NLR) and C-reactive protein (CRP) levels, and other associated laboratory indicators, including plasma albumin, albumin/globulin ratio (AGR), and haemoglobin levels, were included. All parameters were collected on the patient's first day of admission.

***Disease indicators***

The associated disease indicators included tumour characteristics (tumour type, tumour-node-metastasis [TNM] stage, tumour recurrence and metastasis, tumour-bearing status [resected or unresected tumours]), scheduled anti-tumour treatments (ANTs), and comorbidities associated with cachexia (chronic obstructive pulmonary disease [COPD], active tuberculosis, coronary heart disease, myocardial infarction, cerebral stroke, hypertension, diabetes, chronic hepatitis disease, and chronic kidney disease). The diseases occurring rarely in our dataset (<20 cases), including acquired immune deficiency syndrome, inflammatory bowel disease, and severe organ dysfunction (e.g., heart, kidney, and liver failure), were excluded. All comorbidities were determined based on the 10th edition of the international Classification of diseases (ICD-10).[1]

**Table 1.1. Weight loss scale scores of the Patient-Generated Subjective Global Assessment (PG-SGA).**

| **% Weight loss in one month** | **Scores** | **% Weight loss in six months** |
| --- | --- | --- |
| ≥10%  5–9.9%  3–4.9%  2–2.9%  0–1.9% | 4 points  3 points  2 points  1 point  0 point | ≥20%  10–19.9%  6–9.9%  2–5.9%  0–1.9% |
| Any weight loss in the past two weeks | 1 point |  |

**Table 1.2. Quantitative assessment (weighted score) of food intake reduction.**

| **Q**uestions | **Percent of intake reduction** | **Scores** |
| --- | --- | --- |
| The patient had a normal daily diet for the past two weeks. |  | 0 point |
| The patient had a normal but slightly reduced diet compared to his or her daily diet in the past two weeks. | ≤25% | 1 point |
| The patient had a significant decrease in solid food intake in the past two weeks. | ≤50% | 2 points |
| The patient had solid food intake of ≤50% of their daily intake or could only consume a liquid diet in the past two weeks. | >50% | 3 points |
| If the patient is using artificial nutritional preparation, the percent of intake reduction should be adjusted according to the actual nutritional substitution of diet and energy/protein requirements (e.g., partial substitution and full substitution). |  |  |

**Table 1.3. Refined questionnaire scale of physical activity function.**

| **Question** | **Status** | **Score** | **Final score** |
| --- | --- | --- | --- |
| Do you have difficulty carrying heavy objects (approximately 20 kg) or walking long distances (approximately two kilometres)? | No | 0 point | The final score is subject to the highest score. |
|  | Yes | 1 point |  |
| justDo you have difficulty walking short distances (within one kilometres), or do you need to stay in bed or a chair during the day, but not for more than half a day? | No | 0 point |  |
|  | Yes | 2 points |  |
| Do you need help eating, dressing, or bathing, or do you need stay in bed or in a chair most of the day; are you almost completely in bed and unable to get up？ | No | 0 point |  |
|  | Yes | 3 points |  |

**Table 1.4. Refined questionnaire scale for fatigue.**

| **Question** | **Status** | **Score** | **Final score** |
| --- | --- | --- | --- |
| Do you frequently need to rest? | No | 0 point | The final score is the sum of the three questionnaire scores |
|  | Yes | 1 point |  |
| Do you frequently feel weak? | No | 0 point |  |
|  | Yes | 1 point |  |
| Do you frequently feel tired? | no | 0 point |  |
|  | yes | 1 point |  |

**Table 1.5. Refined questionnaire scale for anorexia.**

| **Question** | **Status** | **Score** |
| --- | --- | --- |
| Have you felt loss of appetite in the past month? | No | 1 point |
|  | A bit | 2 points |
|  | Quite | 3 points |
|  | Very | 4 points |


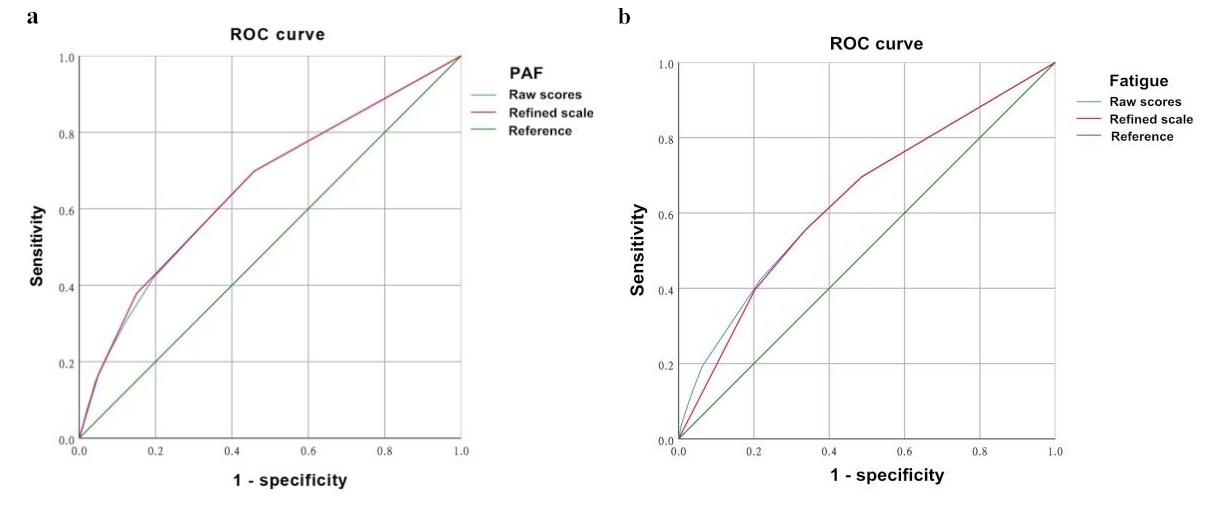


**Figure 1.1. Receiver operator characteristic (ROC) curves for determining the sensitivity and specificity of the refined scales and the raw scores for detecting risk of death.**

**Notes:** The diagnostic value and diagnostic accuracy (sensitivity and specificity) of the refined scales is exactly consistent with the raw scores derived from the EORTC QLQ-C30. The areas under the curves (95% confidence intervals) were (a) 0.655 (0.644, 0.667) vs 0657 (0.646, 0.669) for the refined scale and raw score for PAF domain (p<0.001), and were (b) 0.642 (0.630, 0.654) vs 0.633 (0.622, 0.645) for the refined scale and raw score for fatigue domain (p<0.001).

PAF, physical activity function.

**Section 2. Establishment of the scales**

***Step 1: Screening optimum outcome indicators***

The clinical significance of identification of cachexia lies in identifying cut-offs of nutritional deterioration associated with poor outcomes, and in guiding interventions to improve outcomes. Cachexia may be defined as a component of malnutrition which should be combined with disease characteristics, with irreversible nutrition deterioration progressing until death.[2] Although death risk is not equivalent to cachexia risk, most previous studies used the overall death risk as outcomes for cachexia parameters, using Cox proportional hazards models to generate generating estimated hazard ratios (HRs) and 95% confidence intervals (95%CIs). We also followed this approach and performed survival analyses using the Kaplan–Meier method as compared with log-rank tests.

For domains with multiple available indicators, including domains of the PAF, muscle mass and body composition, and inflammation and catabolic drivers, the receiver operating characteristic (ROC) curves and the respective areas under the curves (AUCs) were used to evaluate the predictive performance of the indicators pertaining to overall death risk and to screen the optimal indicator of each domain. Then, for the domain of the PAF, the E-PAF (E-PAF vs. KPS vs. P-PAF, 0.655 [0.644, 0.667] vs. 0.640 [0.628, 0.651] vs. 0.612 [0.600, 0.624], p˂0.001) showed the best distinct values and was used in the follow-up analysis.

The role of skeletal muscle as key predictor of outcomes and nutritional state is increasingly appreciated.[6, 8] Assessment of skeletal muscle mass based on accurate techniques may however still be too expensive and inconvenient to use in routine clinical practice. Researchers have recommended the easily available BMI or MAMC as alternative indicators for diagnosis of cachexia.[12] In the derivation set, only 2,601 (25.95%) patients underwent BIA analyses. Available data showed that %ASMI and %FFMI had the highest diagnostic value among indicators associated with body composition (AUCs [95%CIs], %ASMI vs. %FFMI vs. BMI vs. %MAMC, 0.623 [0.601, 0.646] vs. 0.605 [0.582, 0.627] vs. 0.597 [0.574, 0.620] vs. 0.537 [0.514, 0.560], *p*˂0.001). However, the FFMI was excluded due to limited availability data. The ASMI was also excluded, because it was obtained by an extremely complex calculation formula,[19] and was inconvenient in the clinical application. Then, we included BMI as a surrogate marker in the final scale, although its changes do not allow to detect changes in body composition.

Most studies take sarcopenia, which is diagnosed according to muscle loss, reduced muscle strength, and PAF decline, as an important basis for the diagnosis of cachexia.[12, 17] Our data demonstrated that the indicators of muscle loss, muscle strength, and PAF decline showed independent effects on death risk, and these variables, including BMI, HGS and E-PAF, were consequently independently included in further analyses.

The inflammation and catabolic drivers are also key indicators for the evaluation and diagnosis of cachexia. In available data, the CRP had slightly higher AUCs than NLR for outcome prediction (AUCs [95% CIs], 0..652 [0.634, 0.669] vs. 0.643 [0.632, 0.655], *p*˂0..001). However, CRP was not used due to incomplete data availability (3,740 (37.31%) of patients in the derivation set). While, the NLR can be obtained using routine blood tests. Then, we included the NLR in the final scale.

In the univariate Cox proportional hazards analysis, there were 11 nutritional/functional indicators, including BMI, HGS, albumin, AGR, NLR and haemoglobin levels, scores of food intake reduction, weight loss, anorexia, fatigue and E-PAF, significantly associated with death risk (*p*˂0.10). Compared with patients with other tumour types, those with pancreatic, lung, liver, gastric, and oesophageal cancer, as well as cholangiocarcinoma, showed higher death risks; while those with breast, endometrial, cervical, bladder, and colorectal cancer, as well as nasopharyngeal carcinoma, showed lower death risks; patients with prostate, bladder, and ovarian cancer showed no significant differences in death risk. Advanced TNM stage, tumour recurrence, an existing unresected focus, and the numbers of organ metastases were found to be risk factors. Scheduled radical ANTs, including radical resection or radiotherapy, was a protective factor. COPD, stroke, and active tuberculosis were associated with a significantly higher death risk; hypertension and diabetes were also associated with a slightly increased death risk, whereas the other commodities showed no significant differences. (Table 2.1).

The 11 nutrition indicators, all tumour types (including prostate, bladder, and ovarian cancers), and the other disease indicators showing a significant difference were included in a multivariate Cox proportional hazards model for evaluating overall death risk. Ten nutrition indicators, including BMI, HGS, albumin, AGR, NLR, scores of the intake loss, weight loss, anorexia, fatigue, and E-PAF, and disease indicators including pancreatic, lung, liver, gastric, oesophageal, breast, and bladder cancer, cholangiocarcinoma, nasopharyngeal carcinoma, TNM stage, unresected focus, the number of metastases, and scheduled radical ANTs showed significant differences. Cervical, prostate, and ovarian cancers, endometrial carcinoma, tumour recurrence, haemoglobin levels, and all comorbidities, including COPD, stroke, active tuberculosis, hypertension, and diabetes, were excluded. (Table 2.1).

***Step 2: Establishment of a nutrition-weighted scoring scale (NWSS)***

***The classifications, cut-off values, and weighted scores of the items in the NWSS***

Consensus suggests that nutrition indicators should be classified in evaluating the severity of cachexia. For both effectiveness and simplicity, indicators were classified into three categories: normal, moderate, and severe. The raw hierarchical variables are four- or five-grade variables, including weight loss, intake reduction, E-PAF, fatigue and anorexia, and their raw grades with similar HRs were combined together into three new classifications. BMI, albumin and AGR levels were characterized using clinical routine cut-off values. NLR and HGS has no clinical routine cut-off values, and were therefore stratified using Youden indices calculated from the ROC curves (NLR) or quartiles by sex and age group for adult men (ages 18–64 years), adult women, elderly men (ages ≥65 years), and elderly women (HGS). It should also be pointed out that establishing scales using equation coefficients directly is not convenient for clinical application. Necessary approximate rounding of weighted scores (the greater the HR, the higher the score assigned) can simplify scales and facilitate clinical implementation, although this may potentially reduce diagnostic accuracy.

Overall, ten optimal nutrition items and their weighted scores were determined to establish the NWSS using a summation method. These items included low albumin and AGR (with the highest weighted scores: 0, 2, 4), followed by anorexia, increased NLR, and intake reduction (0, 2, 3), low BMI and PAF (0, 1, 3), and weight loss, fatigue, and low HGS (with the lowest weighted scores; 0, 1, 2). The process of obtaining the weighted scores for each item is illustrated in Table 2.3. The final NWSS is reported in Manuscript Table 2.

***Classifications of the NWSS (nutrition risk)***

The scores of the NWSS (0–29 points) with similar HRs and survivals were combined into a five-grade classification of nutrition risk (Manuscript Figure 2).[18] The five-grade nutrition risk are summarized for finer categorizations of malnutrition elements in Figure 2.1.

**Table 2.1. Nutritional/functional and disease indicators related to death risk in the univariate Cox proportional hazards analysis.**

| **Variables** | **HRs** | **95% CIs** | **P value** | **Variables** | **HRs** | **95% CIs** | **P value** |
| --- | --- | --- | --- | --- | --- | --- | --- |
| **Breast cancer (yes vs. other cancers)** | 0.186 | 0.158/0.218 | ˂0.001 | **Tumour-node-metastasis (TNM) stage (stage I-IV)** | 2.825 | 2687/2.969 | ˂0.001 |
| **Nasopharyngeal carcinoma (yes vs. other cancers)** | 0.566 | 0.518/0.619 | ˂0.001 | **Distant organ metastasis (numbers, n)** | 1.911 | 1.858/1.966 | ˂0.001 |
| **Endometrial carcinoma (yes vs. other cancers)** | 0.779 | 0.67/0.905 | 0.001 | **Tumour recurrence (yes vs. no)** | 3.469 | 3.242/3.712 | ˂0.001 |
| **Cervical cancer (yes vs. other cancers)** | 0.815 | 0.767/0.865 | ˂0.001 | **Existing focus (yes vs. no)** | 2.555 | 2.347/2.782 | ˂0.001 |
| **Prostate cancer (yes vs. other cancers)** | 0.963 | 0.888/1.043 | 0.354 | **Scheduled radical anti-tumour treatment (yes vs. no)** | 0.380 | 0.352/0.410 | ˂0.001 |
| **Ovarian cancer (yes vs. other cancers)** | 1.012 | 0.978/1.048 | 0.484 | **Total protein (g/L)** | 0.975 | 0.97/0.979 | ˂0.001 |
| **Bladder cancer (yes vs. other cancers)** | 0.983 | 0.933/1.036 | 0.519 | **Albumin (g/L)** | 0.913 | 0.908/0.919 | ˂0.001 |
| **Colorectal cancer (yes vs. other cancers)** | 0.982 | 0.972/0.993 | 0.001 | **AGR (ratio)** | 0.188 | 0.167/0.211 | ˂0.001 |
| **Gastric cancer (yes vs. other cancers)** | 1.03 | 1.02/1.041 | ˂0.001 | **C-reactive protein (mg/L)** | 1.008 | 1.007/1.009 | ˂0.001 |
| **Oesophageal cancer (yes vs. other cancers)** | 1.056 | 1.044/1.069 | ˂0.001 | **NLR (ratio)** | 1.065 | 1.060/1.071 | ˂0.001 |
| **Liver cancer (yes vs. other cancers)** | 1.047 | 1.031/1.064 | ˂0.001 | **Haemoglobin (g/L)** | 0.987 | 0.985/0.988 | ˂0.001 |
| **Lung cancer (yes vs. other cancers)** | 1.075 | 1.069/1.081 | ˂0.001 | **BMI (kg/m^2^)** | 0.91 | 0.900/0.919 | ˂0.001 |
| **Pancreatic cancer (yes vs. other cancers)** | 1.113 | 1.097/1.13 | ˂0.001 | **%MAC (%)** | 0.977 | 0.974/0.98 | ˂0.001 |
| **Cholangiocarcinoma (yes vs. other cancers)** | 1.121 | 1.099/1.144 | ˂0.001 | **%ASMI (%)** | 0.967 | 0.964/0.970 | ˂0.001 |
| **Tuberculosis (yes vs. no)** | 1.729 | 1.058/2.825 | 0.029 | **%MAMC (%)** | 0.991 | 0.988/0.993 | ˂0.001 |
| **Cirrhosis (yes vs. no)** | 0.944 | 0.688/1.294 | 0.72 | **%FFMI (%)** | 1.022 | 1.013/1.031 | ˂0.001 |
| **Chronic hepatitis (yes vs. no)** | 1.151 | 0.99/1.338 | 0.068 | **HGS (kg)** | 0.985 | 0.982/0.988 | ˂0.001 |
| **Cerebral stroke (yes vs. no)** | 1.645 | 1.179/2.295 | 0.003 | **Intake reduction (score)** | 1.801 | 1.722/1.884 | ˂0.001 |
| **COPD (yes vs. no)** | 2.001 | 1.574/2.544 | ˂0.001 | **Weight loss (score)** | 1.275 | 1.247/1.304 | ˂0.001 |
| **Myocardial infarction (yes vs. no)** | 1.114 | 0.646/1.92 | 0.698 | **Anorexia (score)** | 1.805 | 1.731/1.883 | ˂0.001 |
| **Diabetes (yes vs. no)** | 1.164 | 1.037/1.307 | 0.01 | **Fatigue (score)** | 1.23 | 1.211/1.249 | ˂0.001 |
| **Hypertension (yes vs. no)** | 1.092 | 1.002/1.189 | 0.044 | **E-PAF (score)** | 1.122 | 1.113/1.131 | ˂0.001 |
| **Coronary heart disease (yes vs. no)** | 1.158 | 0.992/1.353 | 0.063 | **Karnofsky score (score)** | 0.975 | 0.973/0.976 | ˂0.001 |
| **Chronic kidney disease (yes vs. no)** | 1.666 | 0.895/3.099 | 0.107 | **P-PAF (score)** | 1.624 | 1.569/1.681 | ˂0.001 |

**Notes:** TNM stage, tumour-node-metastasis; AGR, albumin/globulin ratio; NLR, neutrophil/lymphocyte ratio; HGS, handgrip strength; % MAC, percent, mid-arm circumference; % ASMI, percent of appendicular skeletal muscle index; % MAMC, percent, mid-arm muscle circumference; % FFMI, percent, fat free mass index; BMI, body mass index; WL, weight loss; COPD, chronic obstructive pulmonary disease; muscle content, muscle content derived from the bioelectrical impedance analysis; scheduled radical anti-tumour treatment, radical surgery and radiotherapy; P-PAF, physical activity function (P-PAF) of the Patient-Generated Subjective Global Assessment (PG-SGA) score; E-PAF, represented by raw scores for the physical activity function (E-PAF), fatigue, and anorexia domains in the quality-of-life instrument designed by the European Organisation for Research and Treatment of Cancer (EORTC QLQ-C30). Distant organ metastasis (counts), patients with ≥3 organ metastases were recorded as having a (capped) count of 3.

**Table 2.2. Nutrition and disease indicators related to death risk on multivariate Cox proportional hazards modelling.**

| **Disease indicators** | **HRs** | **95% CIs** | **P value** | **Nutritional/functional indicators** | **HRs** | **95% CIs** | **P value** |
| --- | --- | --- | --- | --- | --- | --- | --- |
| **Breast cancer (yes vs. other cancers)** | 0.563 | 0.457/0.692 | ˂0.001 | **Anorexia (score)** | 1.067 | 1.008/1.13 | 0.027 |
| **Nasopharyngeal carcinoma (yes vs. other cancers)** | 0.694 | 0.62/0.777 | ˂0.001 | **Intake reduction (score)** | 1.188 | 1.12/1.259 | ˂0.001 |
| **Bladder cancer (yes vs. other cancers)** | 1.066 | 1.009/1.127 | 0.023 | **Weight loss (score)** | 1.045 | 1.018/1.073 | 0.001 |
| **Colorectal cancer (yes vs. other cancers)** | 1.029 | 1.009/1.049 | 0.004 | **Body mass index (kg/m^2^)** | 0.984 | 0.973/0.995 | 0.003 |
| **Gastric cancer (yes vs. other cancers)** | 1.072 | 1.053/1.091 | ˂0.001 | **Hand grip strength (kg)** | 0.996 | 0.992/0.999 | 0.013 |
| **Oesophageal cancer (yes vs. other cancers)** | 1.099 | 1.08/1.119 | ˂0.001 | **Albumin (g/L)** | 0.979 | 0.971/0.987 | ˂0.001 |
| **Liver cancer (yes vs. other cancers)** | 1.062 | 1.042/1.083 | ˂0.001 | **Albumin/globulin ratio (ratio)** | 0.582 | 0.504/0.674 | ˂0.001 |
| **Lung cancer (yes vs. other cancers)** | 1.064 | 1.052/1.077 | ˂0.001 | **Neutrophil/lymphocyte ratio (ratio)** | 1.031 | 1.024/1.037 | ˂0.001 |
| **Pancreatic cancer (yes vs. other cancers)** | 1.092 | 1.072/1.111 | ˂0.001 | **Fatigue (score)** | 1.023 | 1/1.047 | 0.053 |
| **Cholangiocarcinoma (yes vs. other cancers)** | 1.105 | 1.081/1.13 | ˂0.001 | **Physical activity function (score)** | 1.031 | 1.018/1.045 | ˂0.001 |
| **Existing focus (yes vs. no)** | 1.472 | 1.306/1.659 | ˂0.001 |  |  |  |  |
| **Scheduled radical anti-tumour treatment (yes vs. no)** | 0.614 | 0.543/0.694 | ˂0.001 |  |  |  |  |
| **Tumour-node-metastasis (TNM) stage (stage I-IV)** | 1.844 | 1.722/1.975 | ˂0.001 |  |  |  |  |
| **Distant organ metastasis (numbers, n)** | 1.187 | 1.143/1.232 | ˂0.001 |  |  |  |  |

**Notes:** The physical activity function, fatigue, and anorexia variables represent the raw score of the physical activity function, fatigue, and anorexia domains derived from the quality-of-life instrument designed by the European Organisation for Research and Treatment of Cancer (EORTC QLQ-C30). Cervical, prostate, and ovarian cancer, endometrial carcinoma, tumour recurrence, haemoglobin levels, and all comorbidities, including chronic obstructive pulmonary disease, stroke, active tuberculosis, hypertension, and diabetes, were excluded.The detailed definitions of the scores for weight loss and intake reduction are described in Section 5. Scheduled radical anti-tumour treatments included radical surgery and radiotherapy. Distant organ metastasis (counts), patients with ≥3 organ metastases were recorded as having a (capped) count of 3.

**Table 2.3. Establishment steps (hazard ratios [HRs] and survivals of each category) of the nutrition-weighted scoring scale.**

| **Hierarchical variables, raw scores and grades** | **HRs** | **95% CIs** | **^*^P value** | **Mean survival (months)** | **Median survival (months)** | **^#^P value** | **New adjusting categories (weighted scores)** | **HRs** | **95.0% CI** | **^*^P value** | **Mean survival (months)** | **Median survival (months)** | **^#^P value** |
| --- | --- | --- | --- | --- | --- | --- | --- | --- | --- | --- | --- | --- | --- |
| **^1^Intake reduction** |  | | |  |  |  | **Intake reduction** |  | | |  |  |  |
| **None** | Reference | | | 62.23 | NA | ˂0.001 | **Normal (0 point)** | Reference | | | 62.23 | NA | ˂0.001 |
| **≤25%** | 2.042 | 1.903/2.191 | ˂0.001 | 47.79 | 53.85 |  | **Moderate (2 points)** | 2.042 | 1.903/2.19 | ˂0.001 | 47.79 | 53.85 |  |
| **>25% and ≤50%** | 2.885 | 2.543/3.272 | ˂0.001 | 38.69 | 24.40 |  | **Severe (3 points)** | 3.209 | 2.862/3.598 | ˂0.001 | 36.10 | 20.15 |  |
| **>50%** | 5.534 | 4.406/6.952 | ˂0.001 | 21.15 | 8.29 |  |  |  |  |  |  |  |  |
| **^2^Anorexia** |  | | |  |  |  | **Anorexia** |  | | |  |  |  |
| **0 point** | Reference | | | 59.96 | NA | ˂0.001 | **Normal (0 point)** | Reference | | | 59.96 | NA | ˂0.001 |
| **1 point** | 2.042 | 1.891/2.205 | ˂0.001 | 44.74 | 34.75 |  | **Moderate (2 points)** | 2.044 | 1.893/2.207 | ˂0.001 | 44.71 | 34.39 |  |
| **2 points** | 3.429 | 3.057/3.845 | ˂0.001 | 30.80 | 15.22 |  | **Severe (3 points)** | 3.528 | 3.174/3.921 | ˂0.001 | 30.22 | 14.10 |  |
| **3 points** | 4.14 | 3.278/5.227 | ˂0.001 | 26.23 | 10.19 |  |  |  |  |  |  |  |  |
| **BMI** |  | | |  |  |  | **BMI** |  | | |  |  |  |
| **Obesity** | Reference | | | 66.86 | NA | ˂0.001 | **Normal (0 point)** | Reference | | | 61.20 | NA | ˂0.001 |
| **Overweight** | 1.544 | 1.292/1.845 | ˂0.001 | 59.78 | NA |  | **Moderate (1 point)** | 1.432 | 1.326/1.546 | 1.432 | 53.85 | NA |  |
| **Normal weight** | 2.048 | 1.728/2.429 | ˂0.001 | 53.85 | NA |  | **Severe (3 points)** | 2.585 | 2.325/2.874 | 2.585 | 40.20 | 26.70 |  |
| **Underweight** | 3.699 | 3.073/4.452 | ˂0.001 | 40.20 | 26.70 |  |  |  |  |  |  |  |  |
| **^4^Weight loss** |  | | |  |  |  | **Weight loss** |  | | |  |  |  |
| **0 point** | Reference | | | 60.47 | NA | ˂0.001 | **Normal (0 point)** | Reference | | | 60.47 | NA | ˂0.001 |
| **1 point** | 1.568 | 1.383/1.779 | ˂0.001 | 51.14 | NA |  | **Moderate (1 point)** | 1.576 | 1.446/1.717 | 1.576 | 51.02 | NA |  |
| **2 points** | 1.581 | 1.427/1.751 | ˂0.001 | 50.59 | 73.38 |  | **Severe (2 points)** | 2.259 | 2.091/2.44 | 2.259 | 43.07 | 30.51 |  |
| **3 points** | 2.128 | 1.951/2.322 | ˂0.001 | 44.56 | 34.32 |  |  |  |  |  |  |  |  |
| **4 points** | 2.628 | 2.332/2.963 | ˂0.001 | 38.48 | 22.29 |  |  |  |  |  |  |  |  |
| **^5^Physical activity function** |  | | |  |  |  | **Physical activity function** |  | | |  |  |  |
| **0 point** | Reference | | | 63.88 | NA | ˂0.001 | **Normal (0 point)** | Reference | | | 63.88 | NA | ˂0.001 |
| **1 point** | 1.658 | 1.525/1.804 | ˂0.001 | 55.12 | NA |  | **Moderate (1 point)** | 1.658 | 1.525/1.804 | 1.658 | 55.12 | NA |  |
| **2 points** | 2.961 | 2.695/3.254 | ˂0.001 | 40.56 | 27.75 |  | **Severe (3 points)** | 3.388 | 3.123/3.674 | 3.388 | 37.70 | 21.92 |  |
| **3 points** | 4.143 | 3.743/4.586 | ˂0.001 | 32.92 | 16.83 |  |  |  |  |  |  |  |  |
| **^6^Fatigue** |  | | |  |  |  | **Fatigue** |  | | |  |  |  |
| **0 point** | Reference | | | 62.83 | NA | ˂0.001 | **Normal (0 point)** | Reference | | | 62.85 | NA | ˂0.001 |
| **1 point** | 1.456 | 1.309/1.619 | ˂0.001 | 56.02 | NA |  | **Moderate (1 point)** | 1.573 | 1.445/1.713 | ˂0.001 | 54.53 | NA |  |
| **2 points** | 1.688 | 1.522/1.871 | ˂0.001 | 52.43 | NA |  | **Severe (2 points)** | 2.499 | 2.307/2.707 | ˂0.001 | 44.01 | 33.96 |  |
| **3 points** | 2.497 | 2.305/2.706 | ˂0.001 | 43.99 | 33.96 |  |  |  |  |  |  |  |  |
| **Continuous variable classifications (weighted scores)** | **HRs** | **95% CIs** | **^*^P value** | **Mean survival (months)** | **Median survival (months)** | **^#^P value** | **Continuous variable classifications (weighted scores)** | **HRs** | **95.0% CI** | **^*^P value** | **Mean survival (months)** | **Median survival (months)** | **^#^P value** |
| **^7^Hand grip strength** |  | | |  |  |  | **^9^Neutrophil/lymphocyte ratio** |  | | |  |  |  |
| **Normal, (0 point)** | Reference | | | 60.34 | NA | ˂0.001 | **Normal (0 point)** | Reference | | | 60.71 | NA | ˂0.001 |
| **Moderate (1 point)** | 1.409 | 1.298/1.529 | ˂0.001 | 53.63 | NA |  | **Moderate (2 points)** | 2.119 | 1.962/2.289 | 2.119 | 44.62 | 35.08 |  |
| **Severe (2 points)** | 1.946 | 1.799/2.105 | ˂0.001 | 45.75 | 41.13 |  | **Severe (3 points)** | 2.719 | 2.479/2.984 | 2.719 | 38.48 | 21.83 |  |
| **^8^Albumin** |  | | |  |  |  | **^10^Albumin/globulin ratio** |  | | |  |  |  |
| **Normal, (0 point)** | Reference | | | 59.36 | NA | ˂0.001 | **Normal, (0 point)** | Reference | | | 62.05 | NA | ˂0.001 |
| **Moderate (2 points)** | 2.255 | 2.09/2.433 | ˂0.001 | 41.21 | 26.24 |  | **Moderate (2 points)** | 2.029 | 1.887/2.183 | ˂0.001 | 47.34 | 44.13 |  |
| **Severe (4 points)** | 4.088 | 3.543/4.717 | ˂0.001 | 26.18 | 11.15 |  | **Severe (4 points)** | 3.784 | 3.433/4.17 | ˂0.001 | 31.65 | 15.81 |  |

**Notes:** ^1-6^ Hierarchical variables, the raw scores, or grades for the hierarchical variables, and the detailed definitions of these indicators are shown in Section 5.

^7-10^ The new classifications and their weighted scores for continuous variables, as well as the detailed definitions of these indicators, are shown in Manuscript Table 2.

Different colours represent the following classifications: normal (blue), moderate (yellow), and severe (red).

^*^*P* values for the HRs (Cox proportional hazards model [indicator method])

^#^*P* value for survival (log-rank tests)

NA, not applicable

**Figure 2.1. Proportion of items (malnutrition elements) in classifications of the nutrition-weighted scoring scale (NWSS).**

**Notes:** (a) nutrition risk 0, characterised by an insufficient nutritional reserve, including handgrip strength (HGS) and physical activity function (PAF) decline, but with no or low ongoing loss, including intake reduction (IR) and weight loss (WL); (b) nutrition risk 1, characterised by ongoing IR and WL, low body mass index (BMI), and fatigue (FA), but with no or low hyperinflammation/catabolism and metabolic disorders, including anorexia (AO), increased neutrophil/lymphocyte ratio (NLR), and low albumin (ALB) and albumin/globulin ratio (AGR); (c) nutrition risk 2, characterised by increased hyperinflammation/catabolism and metabolic disorders; (d) nutrition risk 3, characterised by prevailing hyperinflammation/catabolism and metabolic disorders; (e) nutrition risk 4, extreme nutritional deterioration.


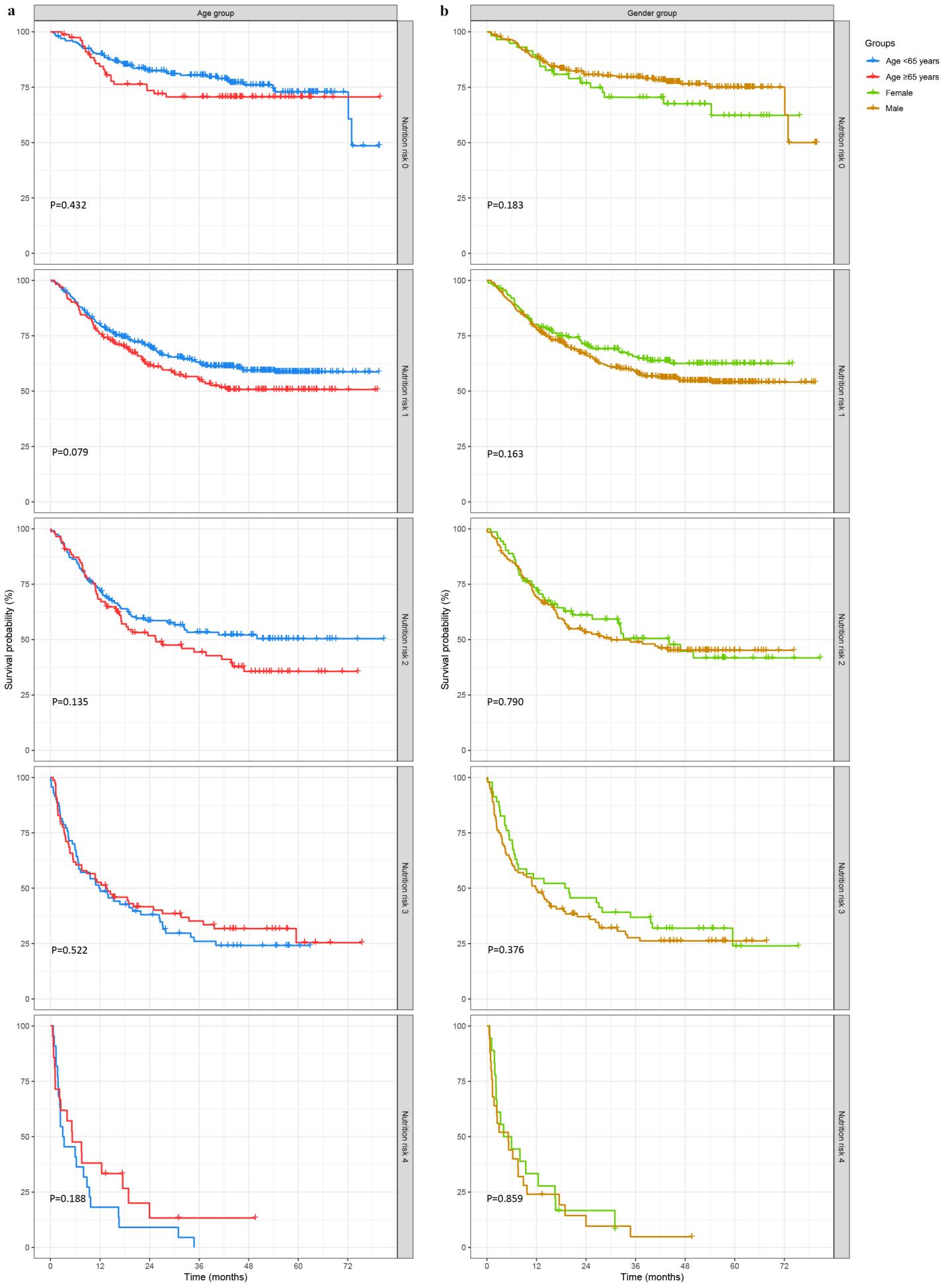


**Figure 2.2. Survival in different subgroups defined according to sex and age in nutrition risk classifications of the nutrition-weighted score scale.**

**Notes:** There were no significantly different in nutrition risk classifications between (a) adult (ages 18–64 years) and elderly patients (ages ≥65 years), and between (b) males and females, in patients with specific tumour types, e.g., gastric cancer.

***Step 3: Establishment of a disease-weighted scoring scale (DWSS)***

Patients with different tumour types and tumour burdens have significant difference in death risk. However, it is difficult to establish a specific scale of disease risk for patients with each tumour type. Therefore, we established a simplified DWSS based on categories of tumour type and tumour burden status for overall patients. Although this method may lead to loss of many details, it can greatly increase the clinical convenience and applicability. Tumour recurrence and all comorbidities were excluded from the multivariate Cox models (Table 2.1 and 2.2) and not included in the DWSS.

***Tumour type***

Overall, 14 tumour types were classified into sequence variables, and Cox proportional hazards analysis was conducted using breast cancer (with the lowest death risk) as the reference. Tumour types with similar HRs and survivals were merged into four categories (weighted score): tumour type Category A (0; with the lowest death risk), including breast and cervical cancer, and nasopharyngeal and endometrial carcinoma; tumour type Category B (2), including prostate, ovarian, bladder, and colorectal cancer; tumour type Category C (4), including gastric, oesophageal, liver cancer, and lung cancer; and tumour type Category D (6; with the highest death risk), including pancreatic cancer and cholangiocarcinoma.

***Tumour burden status***

Chronic hyperinflammatory/catabolism and metabolic disorders induced by an existing tumour is a key pathogenetic factor of cachexia.[9, 11, 12] However, the TNM staging system does not take into account patients’ actual long-term tumour bearing status, and its impact on hyperinflammatory/catabolism and metabolic disorders and nutritional state. Thus, we further established a tumour burden status system based on TNM stage, and radical (existing tumours receiving scheduled radical ANTs or resected tumours receiving adjunct ANTs, without long-term tumour burden) or non-radical tumours (non-radical tumours receiving palliative ANTs or care, with long-term tumour burden), to evaluate patients long-term tumour burden status.

Cachexia is mostly found in patients with advanced stage disease. Nevertheless, we found that many preoperative patients with early stage tumours also had low BMI, weight loss, sarcopenia, and hyperinflammatory/catabolism and metabolic disorders induced by the unresected tumours, but had very well survival and benefited from NT, suggesting that stratification of clinical risk including potential radical treatment is needed for optimal classification and treatment. Thus, in this study, we included patients with radical stage I–II tumours with nutritional derangements. We further found that in patients with stage IV tumours, patients with vital-organ (i.e., lung, live and brain) metastasis or ≥2 distal organ metastases had significantly higher death risk than patients with ≤1 distal organ metastasis and without vital-organ metastasis. We therefore generated eight groups according to TNM stage and radical or non-radical treatment tumour combinations, and performed Cox proportional hazards analysis using patients with Stage I cancer (all radical tumours) as reference. The groups with similar HRs and survivals were combined together into five categories (weighted score), as follows: tumour burden A (0), radical Stage I-II tumours (36 patents with non-radical Stage II tumour were excluded to avoid statistical bias); tumour burden B (1), radical Stage III tumours; tumour burden C (3), including non-radical Stage III tumours and radical Stage IV tumours with ≤1 distal metastasis; tumour burden D (4), including non-radical Stage IV tumours with ≤1 distal metastasis and radical Stage IV tumours with vital-organ metastasis or ≥2 organ metastases; and tumour burden E (5), non-radical Stage IV tumours with vital-organ metastasis or ≥2 organ metastases.

Finally, the DWSS was established using a simple summation method (Manuscript Table 3), which can serve as a completely alternative methodology in reference to complicated matrix methodology (Figure 2.3). The effects of disease characteristics on risks of cachexia are illustrated in notes of the DWSS (Manuscript Table 3).

***Classifications of the DWSS (disease risk)***

We determined a five-grade classification of the DWSS (0–11 points) using the same method as described for the NWSS (Manuscript Figure 2).

***Step 4: Establishment of the lumped scale and categories of comprehensive cachexia risk***

We further established a lumped scale (5×5 matrix) using classifications of both nutrition and disease risks (Manuscript Figure 3),[18] and determined a five grade comprehensive cachexia risk category based on combined evaluation of nutrition and disease risks, death risks and response to NT and ANTs.

**Table 2.4. Establishment steps (hazard ratios [HRs] and survival for each category) for the disease-weighted scoring scale.**

| **Tumour type** | **HRs** (95% CIs） | **^*^*P* value** | **Category** | **Mean survival**  **(95% CIs) (months)** | **Median survival**  **(95% CIs) (months)** | **^#^*P*** value |
| --- | --- | --- | --- | --- | --- | --- |
| **Breast cancer** | Reference |  | **A** | 75.16 (74.11/76.2) | NA (NA) | ˂0.001 |
| **Nasopharyngeal carcinoma** | 0.340 (0.285/0.405) | ˂0.001 | **A** | 69.69 (67.36/72.03) | NA (NA) |  |
| **Endometrial carcinoma** | 0.440 (0.285/0.678) | ˂0.001 | **A** | 68.12 (62.55/73.68) | NA (NA) |  |
| **Cervical cancer** | 0.438 (0.347/0.552) | ˂0.001 | **A** | 68.49 (65.46/71.52) | NA (NA) |  |
| **Prostate cancer** | 0.808 (0.551/1.187) | 0.278 | **B** | 42.94 (36.57/49.31) | 46.70 (NA) |  |
| **Ovarian cancer** | 1.035 (0.844/1.268) | 0.743 | **B** | 52.93 (48.19/57.67) | 60.00 (NA) |  |
| **Bladder cancer** | 0.871 (0.614/1.235) | 0.439 | **B** | 49.98 (42.39/57.56) | NA (NA) |  |
| **Colorectal cancer** | 0.854 (0.774/0.942) | 0.002 | **B** | 57.61 (56.04/59.19) | NA (NA) |  |
| **Gastric cancer** | 1.211 (1.094/1.342) | ˂0.001 | **C** | 49.41 (47.39/51.43) | 72.93 (NA) |  |
| **Oesophageal cancer** | 1.653 (1.459/1.872) | ˂0.001 | **C** | 39.73 (36.69/42.76) | 32.19 (24.97/39.40) |  |
| **Liver cancer** | 1.560 (1.310/1.857) | ˂0.001 | **C** | 42.25 (38.00/46.50) | 38.83 (21.52/56.14) |  |
| **Lung cancer** | 1.847 (1.699/2.008) | ˂0.001 | **C** | 39.59 (37.96/41.22) | 25.64 (23.55/27.74) |  |
| **Pancreatic cancer** | 3.989 (3.301/4.821) | ˂0.001 | **D** | 14.84 (12.64/17.05) | 11.47 (9.74/13.21) |  |
| **Cholangiocarcinoma** | 4.979 (3.819/6.493) | ˂0.001 | **D** | 11.98 (9.39/14.56) | 8.85 (6.58/11.12) |  |
| **Tumour type** category | **HRs** (95% CIs） | **^*^*P* value** | **Weighted score** | **Mean survival (95% CIs) (months)** | **Median survival (95% CIs) (months)** | **^#^**P **value** |
| **Tumour type** category A | Reference |  | **0 point** | 73.02 (72.10/73.95) | NA (NA) | ˂0.001 |
| **Tumour type** category B | 0.766 (0.713/0.824) | ˂0.001 | **2 points** | 57.16 (55.71/58.61) | NA (NA) |  |
| **Tumour type** category C | 1.405 (1.324/1.491) | ˂0.001 | **4 points** | 43.47 (42.32/44.62) | 31.96 (28.85/35.06) |  |
| **Tumour type** category D | 3.719 (3.287/4.208) | ˂0.001 | **6 points** | 13.95 (12.21/15.69) | 10.78 (8.86/12.71) |  |
| **Tumour burden** | **HRs** (95% CIs） | **^*^*P* value** | **Category** | **Mean survival (95% CIs) (months)** | **Median survival (95% CIs) (months)** | **^#^*P* value** |
| **Stage I (all radical tumours)** | Reference |  | **A** | 78.24 (77.21/79.26) | NA (NA) | ˂0.001 |
| **Stage II (all radical tumours)** | 0.281 (0.246/0.320) | ˂0.001 | **A** | 73.40 (72.29/74.52) | NA (NA) |  |
| **Radical Stage III tumours** | 0.679 (0.622/0.742) | ˂0.001 | **B** | 62.61 (61.26/63.96) | NA (NA) |  |
| **Non-radical** Stage III tumours | 1.461 (1.289/1.655) | ˂0.001 | **C** | 47.52 (44.12/50.93) | 39.88 (26.23/53.53) |  |
| **Radical Stage IV tumours with ≤1 distal metastasis** | 1.375 (1.205/1.569) | ˂0.001 | **C** | 48.03 (44.58/51.49) | NA (NA) |  |
| **Non-radical** Stage IV tumours with ≤1 distal metastasis | 1.998 (1.856/2.150) | ˂0.001 | **D** | 39.04 (37.42/40.66) | 27.62 (25.38/29.86) |  |
| **Radical Stage IV with vital-organ metastasis or ≥2 organ metastasis** | 2.686 (2.143/3.368) | ˂0.001 | **D** | 29.22 (22.69/35.75) | 17.98 (5.88/30.09) |  |
| **Non-radical** Stage IV with vital-organ metastasis or ≥2 organ metastasis | 3.841 (3.547/4.160) | ˂0.001 | **E** | 23.17 (21.41/24.93) | 11.93 (10.81/13.06) |  |
| **Tumour burden category** | **HRs** (95% CIs） | **^*^*P* value** | **Weighted score** | **Mean survival (95% CIs) (months)** | **Median survival (95% CIs) (months)** | **^#^*P* value** |
| **Tumour burden A** | Reference |  | **0 point** | 75.45 (74.65/76.25) | NA (NA) | ˂0.001 |
| **Tumour burden B** | 0.616 (0.573/0.662) | ˂0.001 | **1 point** | 62.61 (61.26/63.96) | NA (NA) |  |
| **Tumour burden C** | 1.286 (1.184/1.397) | ˂0.001 | **3 points** | 48.27 (45.81/50.74) | 50.80 (39.52/62.08) |  |
| **Tumour burden D** | 1.830 (1.728/1.938) | ˂0.001 | **4 points** | 38.76 (37.17/40.35) | 27.09 (24.91/29.27) |  |
| **Tumour burden E** | 3.481 (3.264/3.712) | ˂0.001 | **5 points** | 23.15 (21.39/24.90) | 11.93 (10.81/13.06) |  |

**Notes:** The HRs of some tumour type and tumour burden categories were decimals due to the use of the deviation method adjusting for overall survival, with a decimal suggesting lower death risk compared with the overall sample. We multiplied the original HRs by two and rounded them.

Radical tumours, patients receiving or who were planned to receive scheduled radical surgery or radiotherapy; non-radical tumours, patients with unresectable tumours who were receiving or were planned to receive palliative anti-tumour treatments; vital-organ metastasis, including liver, lung, and brain metastasis. Different colours represent significant differences (*P*<0.05) in survival within and between categories (log-rank tests [pairwise comparison]). A total of six categories were determined and were additionally simplified into a four-grade categories for tumour type categories, and a total of seven categories were determined and were additionally simplified into a five-grade categories for tumour burden categories.

Patients with cervical, prostate, ovarian, and bladder cancer, as well as endometrial carcinoma, showed similar survival as compared with the overall sample and showed no significant differences in the Cox proportional hazards analyses due to the use of the deviation method. We then categorised cervical cancer and endometrial carcinoma into tumour type A, and ovarian, prostate and bladder cancer into tumour type B, based on their survival categories (*p*˂0.05) in the Kaplan-Meier analysis as well according to expert opinions.

95% CIs, 95% confidence interval. NA, not applicable

^*^*P* values for the HRs (Cox proportional hazards model [indicator method])

^#^*P* value for survival (log-rank tests)


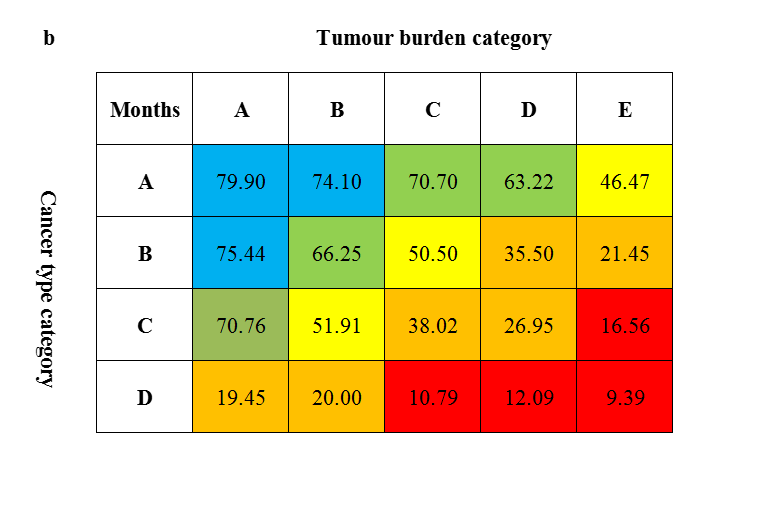

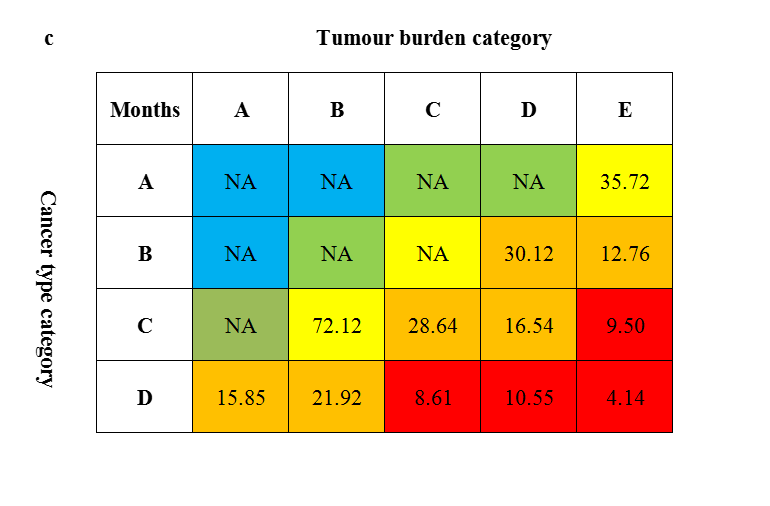

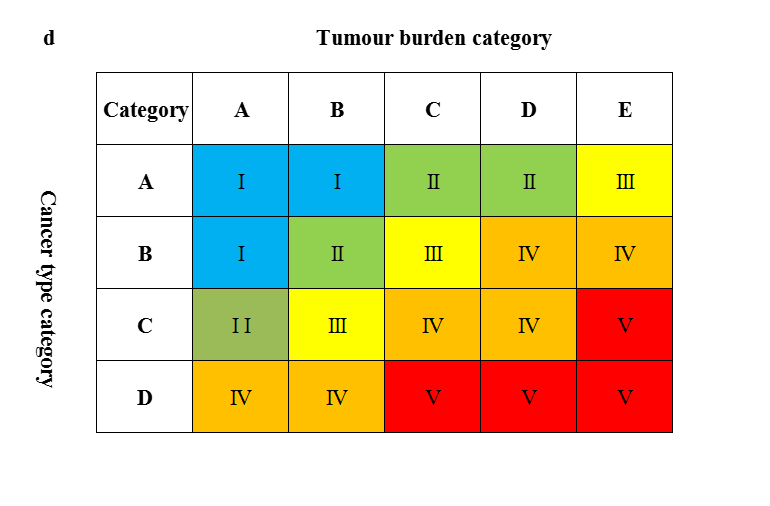


**Figure 2.3. Matrix of the disease-weighted scoring scale (DWSS) combining both tumour type categories and tumour burden status.**

**Notes:** Different colours represent a five-grade classification of the DWSS.

Panels a to d represent a 4×5 matrix analysis of the five-grade classifications according to tumour type and tumour burden category, for a total of 20 combinations. The (a) estimated hazard ratios (HRs), (b) mean survival (months), (c) median survival (months), and (d) categories of disease risk are presented for each cell.

The reference categories represent the lowest tumour type and tumour burden risk.

NA, not applicable.

**Section 3. The difference in phenotype of inflammatory burden/catabolism and metabolic disorders.**

**Figure 3.1. Incidence of the indicators of hyperinflammatory/catabolism and metabolic disorders in different disease characteristics and nutritional status.**

**Notes:** a–b. Intake reduction (IR) and weight loss (WL) were common in patients with malnutrition diagnosed by the Patient-Generated Subjective Global Assessment (PG-SGA) score (Category B and C), which lacked distinguishing value pertaining to non-cachectic malnutrtion (nutrition risk 0–1) and potential cachectic malnutrition (nutrition risk 2–4), while indicators of hyperinflammation/catabolism and metabolic disorders, including anorexia, increased NLR, and low albumin and AGR values were the key to distinguishing non-cachectic malnutrition from cachectic malnutrition. c. patients with unresected tumours (resectable but unresected, and non-radical tumours) had a higher occurrence of hyperinflammation/catabolism and metabolic disorders, especially for an increased NLR, and lower albumin levels, compared with those with resected tumours in each TNM stage, suggesting that there might be a transient inflammatory burden/catabolism and metabolic disorder in preoperative patients, which may be relieved after tumour resection. Patients with unresected stage III tumours evenly had higher NLR, and lower albumin levels than patients with resected stage IV tumours. d. The unresected tumours receiving radical radiotherapy were always local and small size tumours. Patients receiving radical radiotherapy had the lowest occurrence of hyperinflammation/catabolism and metabolic disorders, including the lowest occurrence of lesions of NLR, albumin and anorexia. However, these patients had significantly high occurrence of AGR lesion, these decreased AGR were caused by specificly elevated globin levels alone and might had weak association with systemic inflammation, which deserves further study. e–h. patients with high-risk tumour type, tumour burden status, high disease risk derived from the disease weighted scoring scale, and high comprehensive cachexia risk derived from the lumped scale had increased indicators of hyperinflammation/catabolism and metabolic disorders.

**Section 4. The differential diagnosis of the comprehensive cancer cachexia risk.**

**
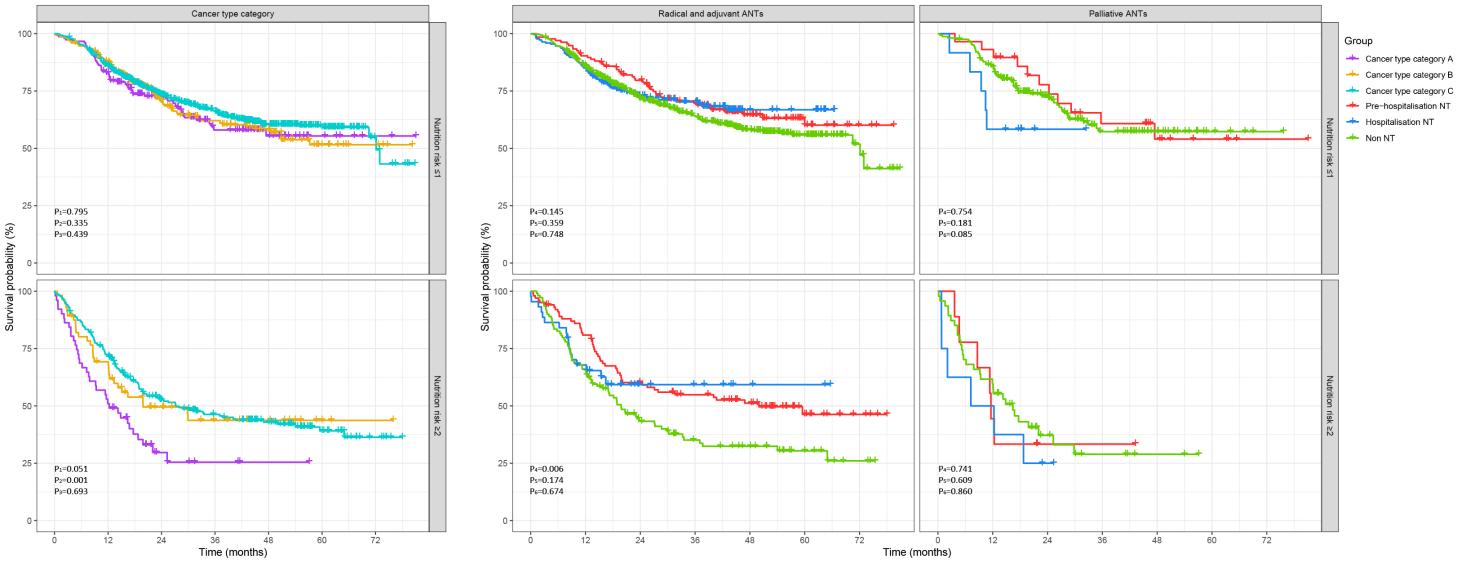
**

**Figure 4.1. The tumour burden heterogeneity and its associated differential diagnosis of the comprehensive cancer cachexia risk.**

**Note:** In the classification of disease risk III by the disease-weighted score scale (DWSS), patients with type A tumours (breast and cervical cancer as well as and nasopharyngeal and endometrial carcinoma) and non-radical Stage IV tumours with vital-organ metastasis or ≥2 organ metastasis, patients with type B tumours (bladder, ovarian, colorectal, and prostate cancers) and non-radical Stage III tumours (25.2%) or radical Stage IV tumours with ≤1 distal metastasis (74.8%), and patients with type C tumours (lung, oesophageal, gastric, and liver cancers) and radical Stage III tumours coexisted. In the nutrition risk 0–1 classification of the nutrition-weighted scoring scale (NWSS), the tumour type heterogeneity had a limited effect on death risk and benefits form nutrition therapy (NT) (upper). In the nutrition risk 2–4 classification of the NWSS, the tumour type heterogeneity caused significant differences in both death risk and NT benefits. Patients with type A tumours and non-radical tumours had a dramatically increased risk of death with increased nutrition risk and benefited less from pre-hospitalisation NT; while patients with higher risk tumour types but radical tumours had a less increased risk of death while still benefiting from pre-hospitalisation NT (lower); suggesting a significant differential diagnosis of the comprehensive cancer cachexia risk associated with tumour burden heterogeneity.

P_1_, type A tumours vs. type B tumours; P_2_ type A tumours vs. type C tumours; P_3_, type B tumours vs. type C tumours; P_4_, pre-hospitalisation NT vs. Non NT; P_5_, hospitalisation NT vs. Non NT; P_6_, pre-hospitalisation NT vs. hospitalisation NT.

**~~
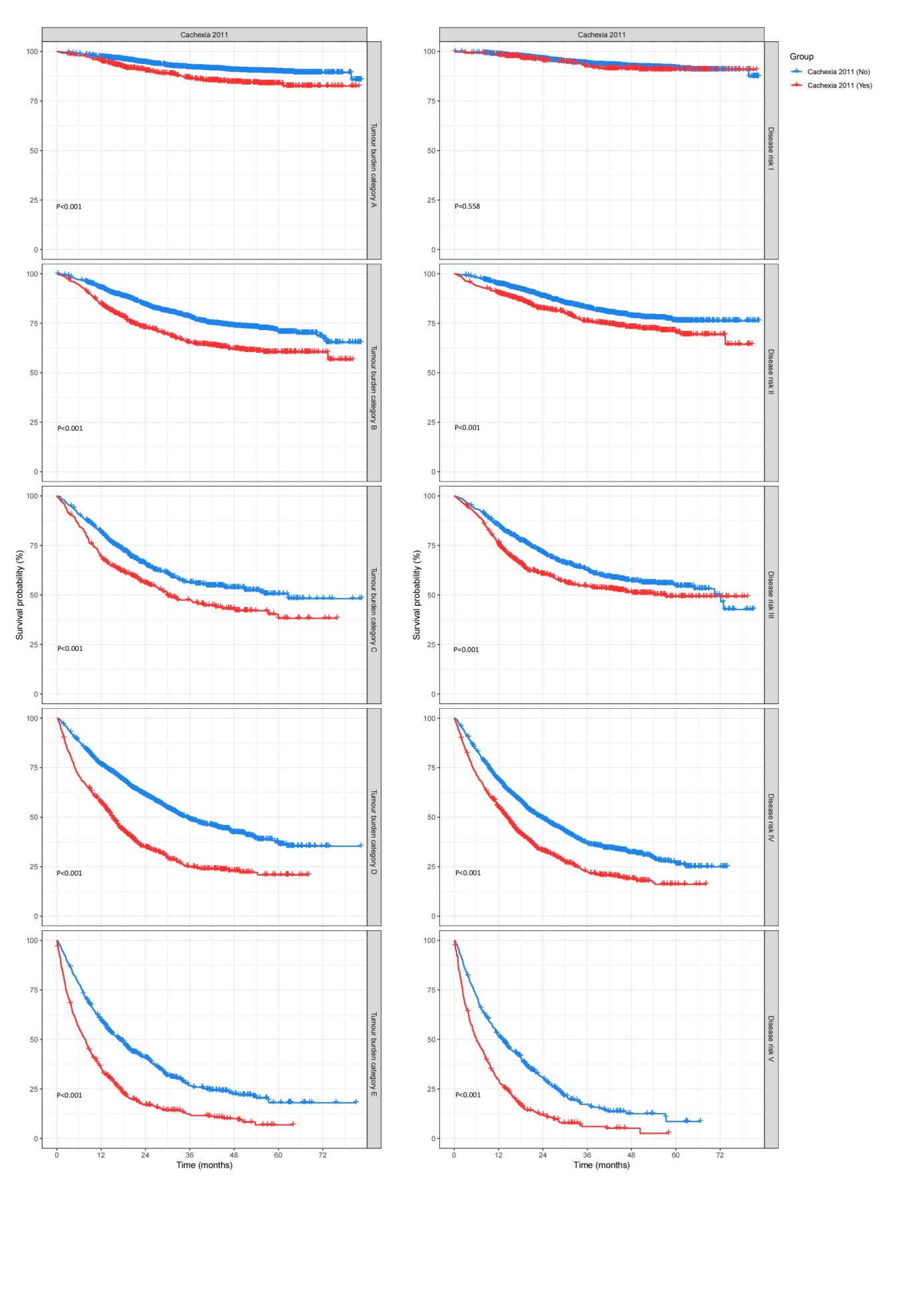
~~**

**Figure 4.2 The survivals of patients with cachexia as per consensus 2011 or not among different tumour burden status and disease risk subgroups**

**Note:** Patients with stage I–II and radical stage III tumour (tumour burden A–B) and disease risk I–II had very low death risk regardless of cachexia as per consensus 2011 or not; patients with tumour burden C and disease risk III had significantly increased death risk regardless of cachexia as per consensus 2011 or not; patients with non-radical stage III and stage IV tumour (tumour burden C–E) and disease risk IV–V, these with cachexia as per consensus 2011 had significant increased death risk than that of patients without cachexia.

~~
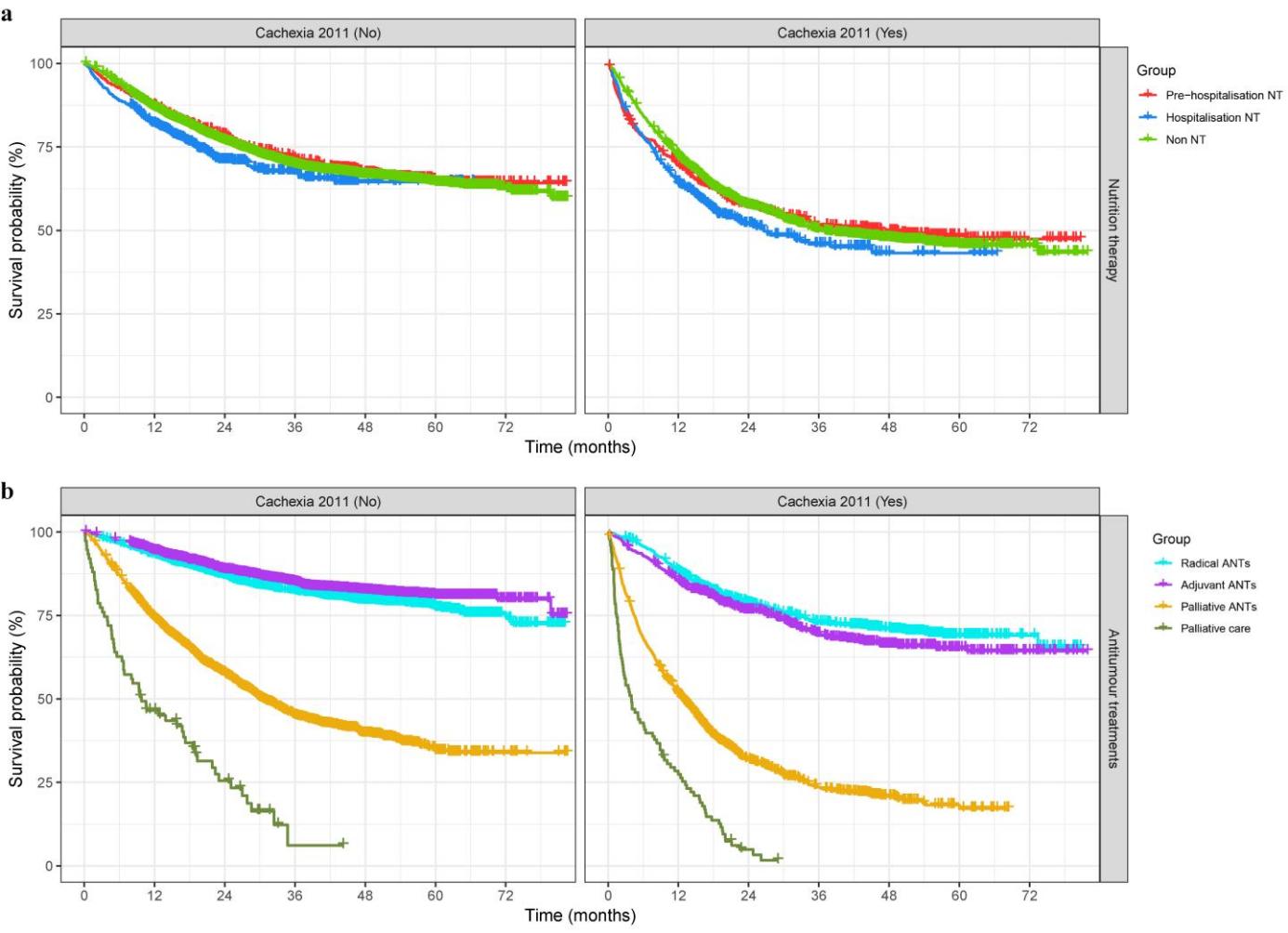
~~

**Figure 4.3 Survival in nutrition therapy (NT) and anti-tumour treatments (ANTs) subgroups in regard to diagnosis as per the consensus 2011.**

**Note:** The classifications of “no cachexia” and “cachexia” as per the Consensus 2011 failed to detect patients who would benefit from (a) NT and (b) ANTs.

**Section 5. The scales yielded consistent distinguishing value regarding a variety of disease characteristics.**

**
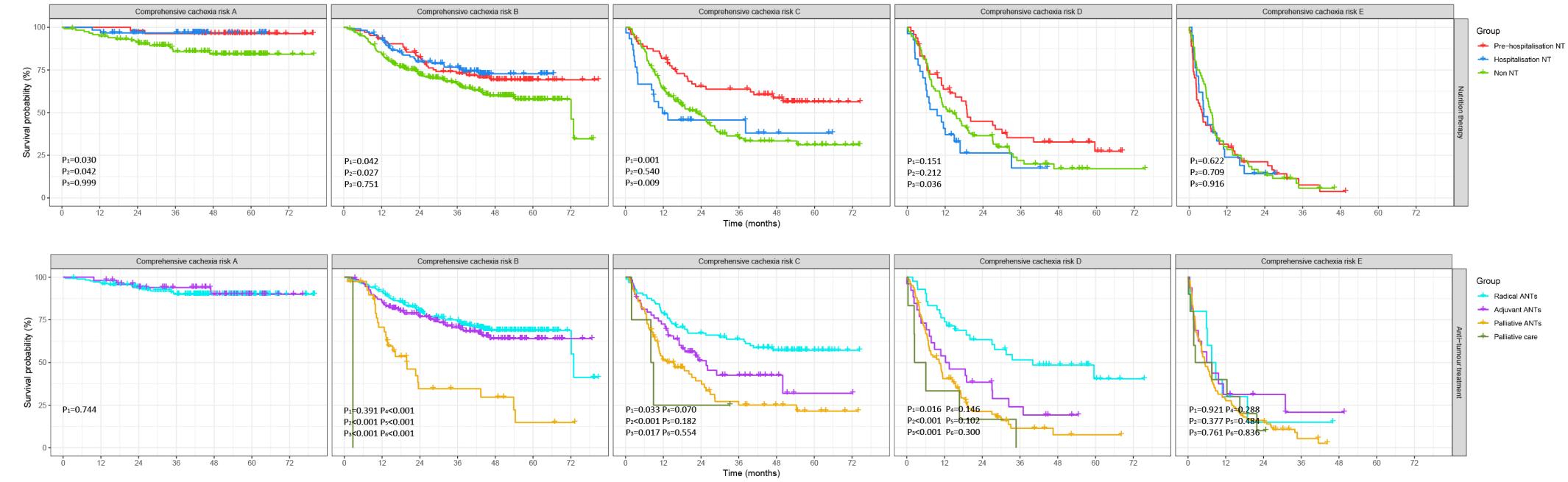
**

**Figure 5.1. Comprehensive risk diagnosis of cachexia in patients with gastric cancer.**

**Note:** Survival in different subgroups according to nutrition therapy (NT) (upper panel) and the anti-tumour treatments (ANTs) (lower panel) in patients with different comprehensive cachexia diagnoses.

In the comprehensive risk A and B categories, most patients (66.39%, n=476/717) with resectable but unresected tumours receiving scheduled radical surgery. Hospitalisation NT comprises a set of routine interventions after radical gastrectomy, therefore, patients receiving hospitalisation NT also had very good survival. However, in the comprehensive risk C and D categories, most patients (65.41%, n=261/399) receiving adjuvant ANTs, or palliative ANTs or care. Hospitalisation NT is not a routine intervention under these circumstances, patients receiving hospitalisation NT had a higher risk of death and poor survival.

Pre-hospitalisation NT (receiving NT before hospitalisation); hospitalisation NT (receiving NT after hospitalisation); and non-NT (not receiving NT).

ANTs, anti-tumour treatments; radical ANTs (radical surgery and radiotherapy), adjuvant ANTs (adjuvant chemotherapy, radiotherapy, molecular targeted therapy, and endocrine therapy after radical surgery), palliative ANTs (palliative surgery, chemotherapy, radiotherapy, molecular targeted therapy, and immunotherapy in patients with unresectable tumour), and palliative care (symptomatic support treatments for relieving pain and improving quality of life).

Upper: P_1_, pre-hospitalisation NT vs. non NT; P_2_, hospitalisation NT vs. non NT; P_3_, pre-hospitalisation NT vs. hospitalisation NT.

Lower: P_1_, radical ANTs vs. adjuvant ANTs; P_2_ radical ANTs vs. palliative ANTs; P_3_, radical ANTs vs. palliative care; P_4_ adjuvant ANTs vs. palliative ANTs; P_5_ adjuvant ANTs vs. palliative care; P_6_ palliative ANTs vs. palliative care

**
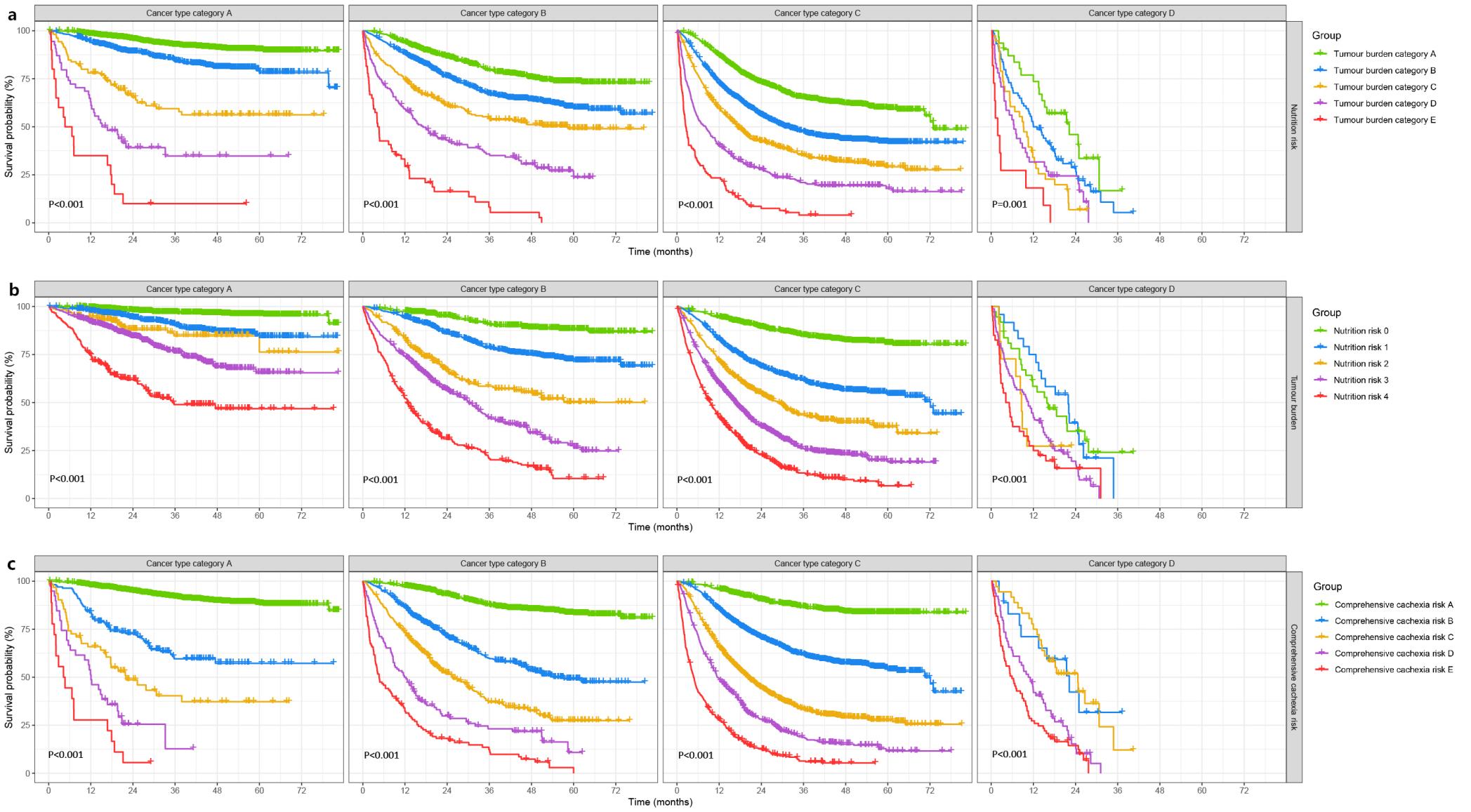
**

**
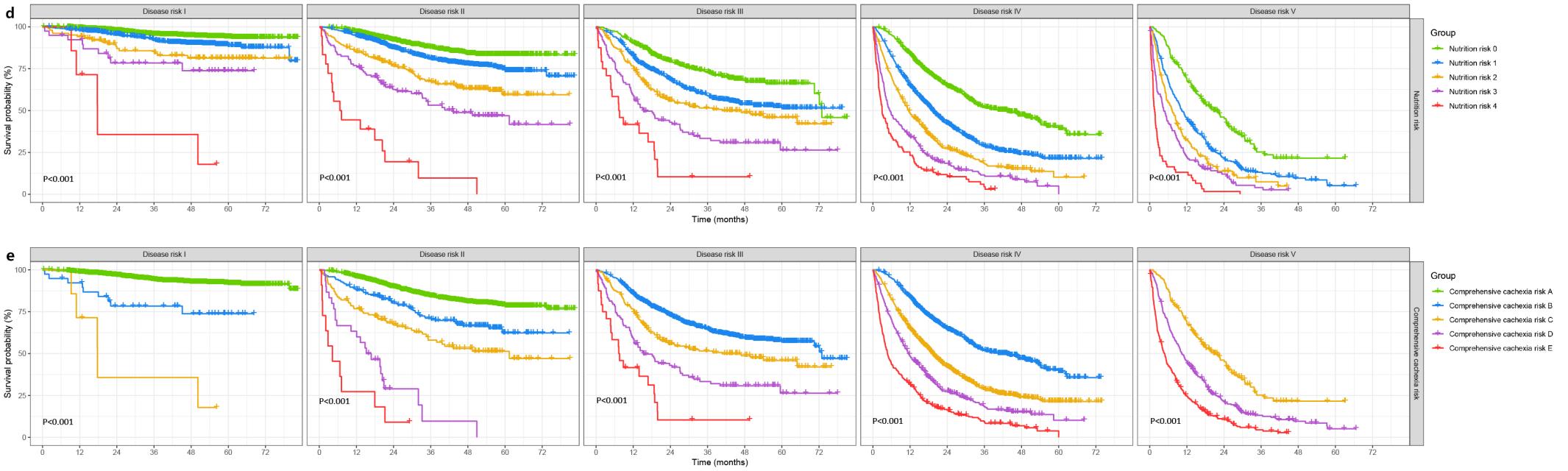
**

**Figure 5.2. The new scales yielded consistent distinguishing value regarding a variety of disease characteristics.**

**Notes:** (a) the tumour burden status categories yield well distinguishing values in patients with different tumour types categories belong to the disease-weighted scoring scale (DWSS); (b) the grading classification (nutrition risk) of the nutrition-weighted scoring scale (NWSS) and (c) the grading classification (comprehensive cachexia risk) of the lumped scale yielded well distinguishing values in patients classified into different tumour type categories; (d) the grading classification of nutrition risk and (e) comprehensive cachexia risk yielded well distinguishing values in patients classified into different disease risks.

Tumour type A, breast and cervical cancer as well as nasopharyngeal and endometrial carcinoma; tumour type B, bladder, ovarian, colorectal, and prostate cancer; tumour type C, lung, oesophageal, gastric, and liver cancer; tumour type D, pancreatic cancer, and cholangiocarcinoma. Tumour burden A, radical Stage I–II tumours; tumour burden B, radical Stage III tumours; tumour burden C, including non-radical Stage III tumours and radical Stage IV tumour with ≤1 distal metastasis; tumour burden D, including non-radical Stage IV tumours with ≤1 distal metastasis or radical Stage IV tumours with vital-organ metastasis or ≥2 organ metastases; tumour burden E, non-radical Stage IV tumours with vital-organ metastasis or ≥2 organ metastases.

**Figure 5.3. Time-dependent receiver operator characteristic curves for determining the sensitivity and specificity of the different scales for detecting mortality risk.**

**Note:** At each time node, the predictive values (areas under the curves [AUCs]) of the nutrition-weighted scoring scale (NWSS), the disease-weighted scoring scale (DWSS), and the lumped scale on the risk of death in the validation set all reached ≥90% of the values derived using the corresponding scale in the derivation set.

**References:**

1.Administrative simplification: adoption of a standard for a unique health plan identifier; Addition to the national provider identifier requirements; And a change to the compliance date for the international classification of diseases, 10th edition (icd-10-cm and icd-10-pcs) medical data code sets. Final rule. Fed Regist. 2012;77:54663-54720.

2.Aapro M, Arends J, Bozzetti F, Fearon K, Grunberg S M, Herrstedt J, et al. Early recognition of malnutrition and cachexia in the cancer patient: a position paper of a european school of oncology task force. Ann Oncol. 2014;25:1492-1499.

3.Aaronson N K, Ahmedzai S, Bergman B, Bullinger M, Cull A, Duez N J, et al. The european organization for research and treatment of cancer qlq-c30: a quality-of-life instrument for use in international clinical trials in oncology. Jnci-J Natl Cancer I. 1993;85:365-376.

4.Anandavadivelan P, Lagergren P. Cachexia in patients with oesophageal cancer. Nat Rev Clin Oncol. 2016;13:185-198.

5.Baracos V E, Martin L, Korc M, Guttridge D C, Fearon K. Cancer-associated cachexia. Nat Rev Dis Primers. 2018;4:17105.

6.Barazzoni R, Jensen G L, Correia M, Gonzalez M C, Higashiguchi T, Shi H P, et al. Guidance for assessment of the muscle mass phenotypic criterion for the global leadership initiative on malnutrition (glim) diagnosis of malnutrition. Clin Nutr. 2022;41:1425-1433.

7.Barton M K. Cancer cachexia awareness, diagnosis, and treatment are lacking among oncology providers. Ca-Cancer J Clin. 2017;67:91-92.

8.Bozzetti F. Forcing the vicious circle: sarcopenia increases toxicity, decreases response to chemotherapy and worsens with chemotherapy. Ann Oncol. 2017;28:2107-2118.

9.Cederholm T, Barazzoni R, Austin P, Ballmer P, Biolo G, Bischoff S C, et al. Espen guidelines on definitions and terminology of clinical nutrition. Clin Nutr. 2017;36:49-64.

10.Chen L K, Woo J, Assantachai P, Auyeung T W, Chou M Y, Iijima K, et al. Asian working group for sarcopenia: 2019 consensus update on sarcopenia diagnosis and treatment. J Am Med Dir Assoc. 2020;21:300-307.

11.Fearon K, Arends J, Baracos V. Understanding the mechanisms and treatment options in cancer cachexia. Nat Rev Clin Oncol. 2013;10:90-99.

12.Fearon K, Strasser F, Anker S D, Bosaeus I, Bruera E, Fainsinger R L, et al. Definition and classification of cancer cachexia: an international consensus. Lancet Oncol. 2011;12:489-495.

13.Gurney J M, Jelliffe D B. Arm anthropometry in nutritional assessment: nomogram for rapid calculation of muscle circumference and cross-sectional muscle and fat areas. Am J Clin Nutr. 1973;26:912-915.

14.Hu F J, Liu H, Liu X L, Jia S L, Hou L S, Xia X, et al. Mid-upper arm circumference as an alternative screening instrument to appendicular skeletal muscle mass index for diagnosing sarcopenia. Clin Interv Aging. 2021;16:1095-1104.

15.Jin M, Du H, Zhang Y, Zhu H, Xu K, Yuan X, et al. Characteristics and reference values of fat mass index and fat free mass index by bioelectrical impedance analysis in an adult population. Clin Nutr. 2019;38:2325-2332.

16.Kim J Y, Wie G A, Cho Y A, Kim S Y, Kim S M, Son K H, et al. Development and validation of a nutrition screening tool for hospitalized cancer patients. Clin Nutr. 2011;30:724-729.

17.MacDonald N. Terminology in cancer cachexia: importance and status. Curr Opin Clin Nutr. 2012;15:220-225.

18.Martin L, Senesse P, Gioulbasanis I, Antoun S, Bozzetti F, Deans C, et al. Diagnostic criteria for the classification of cancer-associated weight loss. J Clin Oncol. 2015;33:90-99.

19.Wen X, Wang M, Jiang C M, Zhang Y M. Anthropometric equation for estimation of appendicular skeletal muscle mass in chinese adults. Asia Pac J Clin Nutr. 2011;20:551-556.

20.Zhou B. [prospective study for cut-off points of body mass index in chinese adults]. Zhonghua Liu Xing Bing Xue Za Zhi. 2002;23:431-434.
